# Testing Green Tea Extract and Ammonium Salts as Stimulants of Physical Performance in a Forced Swimming Rat Experimental Model

**DOI:** 10.3390/ijms251910438

**Published:** 2024-09-27

**Authors:** Ekaterina A. Korf, Artem V. Novozhilov, Igor V. Mindukshev, Andrey S. Glotov, Igor V. Kudryavtsev, Ekaterina V. Baidyuk, Irina A. Dobrylko, Natalia G. Voitenko, Polina A. Voronina, Samarmar Habeeb, Afrah Ghanem, Natalia S. Osinovskaya, Maria K. Serebryakova, Denis V. Krivorotov, Richard O. Jenkins, Nikolay V. Goncharov

**Affiliations:** 1 Sechenov Institute of Evolutionary Physiology and Biochemistry, Russian Academy of Sciences, pr. Torez 44, St. Petersburg 194223, Russia; 2 D.O. Ott Research Institute of Obstetrics, Gynecology and Reproductology, St. Petersburg 199034, Russia; 3 Institute of Experimental Medicine, St. Petersburg 197022, Russia; 4 Research Institute of Hygiene, Occupational Pathology and Human Ecology of the Federal Medical Biological Agency, p.o. Kuz’molovsky bld.93, St. Petersburg 188663, Russia; 5 Leicester School of Allied Health Sciences, De Montfort University, The Gateway, Leicester LE1 9BH, UK

**Keywords:** forced swimming, endurance, rats, m. soleus, m. extensor digitorum longus, green tea extract, ammonium salts, calcium balance, gene expression, morphometry, biochemical and immunological parameters

## Abstract

The study of drugs of natural origin that increase endurance and/or accelerate recovery is an integral part of sports medicine and physiology. In this paper, decaffeinated green tea extract (GTE) and two ammonium salts—chloride (ACL) and carbonate (ACR)—were tested individually and in combination with GTE as stimulants of physical performance in a forced swimming rat experimental model. The determined parameters can be divided into seven blocks: functional (swimming duration); biochemistry of blood plasma; biochemistry of erythrocytes; hematology; immunology; gene expression of slow- and fast-twitch muscles (m. soleus, SOL, and m. extensor digitorum longus, EDL, respectively); and morphometric indicators of slow- and fast-twitch muscles. Regarding the negative control (intact animals), the maximum number of changes in all blocks of indicators was recorded in the GTE + ACR group, whose animals showed the maximum functional result and minimum lactate values on the last day of the experiment. Next, in terms of the number of changes, were the groups ACR, ACL, GTE + ACL, GTE and NaCl (positive control). In general, the number of identified adaptive changes was proportional to the functional state of the animals of the corresponding groups, in terms of the duration of the swimming load in the last four days of the experiment. However, not only the total number but also the qualitative composition of the identified changes is of interest. The results of a comparative analysis suggest that, in the model of forced swimming we developed, GTE promotes restoration of the body and moderate mobilization of the immune system, while small doses of ammonium salts, especially ammonium carbonate, contribute to an increase in physical performance, which is associated with satisfactory restoration of skeletal muscles and the entire body. The combined use of GTE with ammonium salts does not give a clearly positive effect.

## 1. Introduction

The study of the mechanisms of action of drugs of natural origin that increase endurance and reduce fatigue is one of the most important areas of sports physiology and medicine. Nutraceuticals (dietary supplements and food components, minerals and metabolites of natural origin) can increase physical performance and reduce fatigue by positively influencing the balance of signaling and metabolic processes in cells and tissues of the body [1]. The actions of a number of nutraceuticals, which include green tea extract (GTE), are associated with increased fat catabolism, increased insulin sensitivity and glucose tolerance [2]. These and other effects can contribute to an increase in the endurance of animals in experimental models of running and swimming loads [3]. GTE has been shown to be effective in chronic fatigue syndrome [4] and Duchenne muscular dystrophy [5]; the immunomodulatory properties of GTE have been established during intense physical exertion [6].

The peak of GTE popularization, composition research and ingredient characterization dates back to the end of the last and the beginning of this century. It was found that dried green tea contains 15–20% polyphenols (catechins), 25–40% of which are epigallocatechin-3-gallate (EGCG, see Supplementary Tables S1–S3 and Figure S1); in GTE preparations intended for use as a food additive, the EGCG content reaches more than 90% [7]. The pharmacokinetics of EGCG, like other catechins, has been studied in experiments on rats, mice and humans. With a single intragastric administration of radioactively labeled EGCG to rodents [8]: 10% was detected in the blood, and about 1% was detected in the prostate gland, heart, lungs, liver, kidneys and other tissues after 24 h. The main route of excretion of the drug was with feces. In rats, 77% of the intravenously administered dose of EGCG was excreted with bile, while 2% was excreted with urine. The absolute bioavailabilities of EGCG, epigallocatechin (EGC) and epicatechin (EC) with intragastric administration of decaffeinated green tea in rats were 0.1%, 14% and 31%, respectively [9]. Several studies have examined the bioavailability of green tea and its catechins following oral administration in humans. Following peroral administration of green tea powder at a dose of 20 mg/kg body weight, the following plasma Cmax values for EGC, EC and EGCG were found to be 223, 124 and 77.9 ng/mL, respectively [10]. Tmax was determined in the range of 1.3 to 1.6 h, with t_1/2_ being 3.4, 1.7 and 2 h for EGCG, EGC and EC, respectively [11]. In plasma, EC and EGC are present in their conjugated forms, while 77% of EGCG is in its free form [10].

The use of GTE in forced swimming tests leads to increased endurance of rats due to the additional participation of slow-twitch muscles in the work, the adaptation of which is associated with an increase in the expression of genes responsible for regulating the balance of Ca^2+^ ions [12,13]. These data indicate the presence of adaptive mechanisms of regulation of Ca^2+^ ion levels. However, it is important to understand how green tea catechins affect different links in the regulation of calcium balance in slow- and fast-twitch muscle fibers during intense exercise, and in animals adapted to exercise. It is known that the short-term effect of green tea catechins is associated with the inhibition of catechol-*O*-methyltransferase, which, in synergy with caffeine, leads to an increase in the level of catecholamines and cAMP, increased lipolysis and thermogenesis. Long-term effects of catechins are mediated by signaling regulators PGC-1α (PPAR-γ coactivator 1-α) and PPARα,β/δ,γ; they are associated with an increase in the expression of genes responsible for the utilization of fatty acids [14]. In addition, catechins inhibit UDP-glucuronosyltransferase, indirectly regulating testosterone levels [15].

Among the biochemical adaptation mechanisms that are in reciprocal relationships with Ca^2+^ ions, the processes of ATP generation and consumption are of decisive importance under conditions of both aerobic and anaerobic loads. Lactate and ammonia were previously considered metabolic by-products, transported and neutralized exclusively in the liver by the Cori and alanine cycles. The role of lactate was reassessed a long time ago and now it is considered as the most important intercellular energy shuttle and signaling agent [16]. In fast-twitch muscles, the expression of the type 4 monocarboxylate transporter (MCT4) increases for increased export of lactate, which enters erythrocytes and slow-twitch muscles through MCT1 [17]. Red blood cells help transport lactate from producer cells to consumer cells, and the role of red blood cells in trained athletes is increased [18]. However, the attitude towards ammonia as an exclusively toxic agent has hardly changed over the past decades. Ammonia is continuously formed in all organs and tissues of the body. Its most active producers are organs with a high metabolism of amino acids and biogenic amines, nervous tissue, the liver, intestines, and muscles. The main biochemical sources of ammonia are: the oxidative deamination of glutamate in all tissues except muscle; the non-oxidative deamination of some amino acids in the liver; the deamidation of glutamine and asparagine; the catabolism of biogenic amines; the vital activity of colon bacteria; and the breakdown of purine and pyrimidine bases with the participation of adenylate kinase and AMP deaminase. Ammonia is an extremely toxic compound, and therefore in the tissues there are reactions of binding (neutralization) of ammonia through the formation of glutamate, glutamine, asparagine and carbamoyl phosphate. However, it should be understood that in muscles, ammonia generation could be considered not only as a result of amino acid catabolism as an energy source, but also as a compensatory mechanism for hydrogen ion binding and the neutralization of organic acids (primarily lactate), the level of which increases during exercise, disturbing the ionic balance. Unlike untrained volunteers, an increase in the endurance of trained athletes is associated not so much with an increase in VO2max and the oxidative capacity of muscles, but with adaptive changes in the system of regulation of the balance (homeostasis) of Ca^2+^, K^+^, Na^+^, Cl^−^, H^+^ and lactate ions [19]. The role of ammonia in this system of adaptive changes is very poorly understood, although it is clear that excess ammonia is bad, and the use of, for example, arginine and citrulline (components of the urea cycle) in order to neutralize ammonia formed during the utilization of branched-chain amino acids, can increase the endurance of runners by long distances [20]. The effect of small amounts of ammonia/ammonium on physical performance has not been studied extensively. Previously, we conducted a comparative analysis of the effects of GTE and ammonia, which in the form of an ammonium chloride solution (ACL, NH_4_Cl) was tested on its own (10 mg/kg) and in combination with GTE as a stimulant of physical performance. In a model of forced swimming, the stimulating effect of ACL was established, which exceeds the effect of GTE [21]. Multidirectional adaptive changes in some biochemical parameters of blood plasma and erythrocytes of rats were revealed with the use of GTE or ACL, as well as an increase in the effect of these nutraceuticals on the duration of the swimming load when used together [22]. At the same time, it should be noted that there was no imbalance in the indicators of adaptive immunity, which was characteristic of animals in the control group [23]. In experiments with human volunteers, low-dose ammonium preconditioning enhanced endurance in submaximal physical exercises [24].

The purpose of the research reported here was to conduct a comprehensive comparative analysis of biochemical, hematological, immunological and morphometric parameters, together with the expression of genes responsible for the balance of calcium ions in the fast- and slow-twitch skeletal muscles of rats after a cycle of forced swimming, with the use of GTE and small doses of two ammonium salts—ACL (20 mg/kg when used alone and 10 mg/kg in combination with GTE) and ammonium carbonate (ACR, 10 mg/kg both when used alone and in combination with GTE). We proceeded with the assumption that ammonia—as one of the end products of catabolism affecting the ionic balance of cells and the acid–base state of the body, freely penetrating into erythrocytes and other cells—when introduced by small doses through the gastrointestinal tract shortly before performance of aerobic–anaerobic exercise, can contribute to the adaptive rearrangement of metabolic pathways mainly through the expression of genes responsible for the balance of Ca^2+^ ions under conditions of extreme physical activity, thereby increasing the body’s endurance.

## 2. Results

### 2.1. Functional Parameters and Blood Lactate Level after Exercise

In rats of the positive control group (NaCl), the duration of swimming on the last day of the experiment, as well as the average duration of swimming for 4 days of maximum load, did not change significantly in relation to the initial level (Table 1). Rats treated with GTE also did not show a statistically significant increase in endurance.

In the ACL group, the average duration of swimming increased by 31%, and the duration of swimming on the last day of exercise increased by 1.6-fold compared with the NaCl group. An increase in both the duration of swimming on the last day of the experiment and the average duration of swimming was also shown by rats of the ACR group; 1.8- and 1.9-fold, respectively. Rats treated with GTE in combination with ACL showed an increase in swimming time only on the last day (by 40%). In the GTE + ACR group, the duration of swimming on the last day increased 1.9-fold, and their average duration of swimming increased 1.6-fold compared with the positive control.

The concentration of lactate in the blood of rats from the negative control group was 3.6 (2.4; 5.2) mmol/L (Table 2). Five minutes after the load, the concentration of lactate in the blood of rats of all experimental groups was significantly increased, and a maximum two-fold increase in the concentration of lactate was noted in animals from the positive control group. The smallest increases were registered in the ACR group (by 58%) and GTE + ACR group (by 61%). In the groups taking GTE, ACL alone and GTE in combination with ACL, there was a significant increase in lactate concentration by 75%, 72% and 69%, respectively. An hour after the end of the load, the concentration of lactate in the blood of all groups of experimental animals reduced to the level of the negative control, while some groups even showed lower levels.

### 2.2. Biochemical Parameters of Blood Plasma on the Next Day after Exercise

Biochemical parameters of rat blood plasma one day after the end of the forced swimming cycle are presented in Table 3. The concentration levels of total protein, albumin, glucose, free fatty acids, nitrite and nitrate, as well as the activities of ALT, alkaline phosphatase and GPx3 did not show significant differences between the groups. In animals of the ACR group, the concentration of lactate was reduced by 20%, urea by 22% and uric acid by 21% relative to the negative control (intact rats). In rats of the GTE + ACR group, the concentration of creatinine was reduced by 10%, transferrin by 13%, triglycerides by 33%, cholesterol by 20% and high-density lipoprotein by 35% relative to the negative control. At the same time, the concentration of low-density lipoproteins in rats of the GTE + ACR group was reduced by 30% relative to rats in the positive control group (NaCl). The concentration of creatinine relative to the negative control group was also reduced in the NaCl groups by 12% and GTE + ACL by 7%. In the blood plasma of rats of the GTE + ACL group, the phosphorus concentration was increased by 20% relative to the positive control.

In general, the number and nature of changes in biochemical parameters indicate: the absence of significant adaptive changes in the ACL and GTE groups; the minimum number of changes in the NaCl and GTE + ACL groups; the average number of changes (3) in the ACR group; and the maximum number of changes (6) relative to the control in the GTE + ACR group. Taking into account the main functional indicator—swimming duration—the highest result with the lowest cost of biochemical adaptation was achieved in rats of the ACL group, while the indicators of the GTE + ACR and ACR groups indicate more or less significant adaptive shifts. In the ACR group, these changes are associated primarily with increased liver function in the utilization of lactate and urea and a decrease in the production of uric acid, as well as skeletal muscles, which are involved in the utilization of lactate and the production of uric acid [25]. In the GTE + ACR group, biochemical changes are associated with increased lipid catabolism (presumably in slow muscles) and a decrease in their synthesis in the liver. Increased utilization of lipids by skeletal muscles probably causes a decrease in their production of creatinine, which was noted in experiments using another known polyphenol, resveratrol [26].

### 2.3. Biochemical Parameters of Red Blood Cells on the Next Day after Exercise

Of the 24 parameters of red blood cells studied in our experiments, more than half, i.e., 13, did not undergo significant changes in any of the experimental groups relative to the negative or positive control. These parameters include the concentrations of total and reduced glutathione, metHb and ATP, and the activity of the enzymes catalase, glutathione peroxidase, glutathione-S-transferase, lactate dehydrogenase, glucose-6-phosphate dehydrogenase (G6PD), Mg^2+^-ATPase, Ca^2+^-ATPase, Na^+^/K^+^-ATPase and 5′-nucleotidase. Among the experimental groups of animals, only the GTE group did not reveal significant changes in any of the studied biochemical parameters of erythrocytes (Table 4). In the positive control group, a decrease in pyruvate concentration by 14% was detected relative to its level in the negative control group. In rats of the ACL group, the concentration of MDA was reduced both relative to the negative control group (by 26%) and relative to the positive control group (by a third). In addition, in the ACL group the GSH/GSSG ratio was increased by 43% relative to the positive control group. In rats of the ACR group, relative to the intact control, the lactate concentration was reduced by 21%, GR activity by 31%, transmembrane electron transport (TMET) by 1.9-fold, and relative to the positive control, the GSSG concentration was reduced by 42%. In rats of the GTE + ACL group, relative to the intact control, the 2,3-bisphosphoglycerate (BPG) concentration was increased by 28%, the lactate concentration was decreased by 23%, GAPD activity was decreased by 10%, and GR activity was decreased by 28%. At the same time, relative to the positive control group, the GSH/GSSG ratio increased by 80% and the level of pyruvate increased by 8%.

The dynamics of MDA seem to be the most paradoxical: this value for the ACL group decreases compared to both controls, and curiously this effect does not occur in co-supplementations with GTE. It is noteworthy that the significant decrease in the median is due to a striking decrease in the upper limit of the range and, as a consequence, the minimum range of MDA values in the ACL group rats. Consequently, either there is an increased renewal of the erythrocyte pool, or the mechanism for removing MDA from cells is involved, or there is a combination of both mechanisms. It is known that the lifespan of rat erythrocytes is two-fold shorter than that of humans [27,28,29]. When exposed to toxic factors and certain diseases, and also when adapting to physical activity, the lifespan of erythrocytes is significantly reduced, especially in model experiments on rats [30,31,32,33]. Not surprisingly that the level of MDA in the fraction of young erythrocytes is significantly lower than in the fraction of old erythrocytes [29]. As for the mechanism of removal of lipid peroxidation products from cells, it is known, for example, that the inactivation of 4-hydroxy-2-nonenal (HNE) occurs through glutathione-S-transferases (GST) with the formation of a water-soluble conjugate GS-HNE [34]. Importantly, the resulting conjugate is a strong inhibitor of GST and must be removed from the cell so as not to interfere with the enzyme and neutralize HNE. About 3/4 of the conjugate is transported from the cell by the RLIP76 protein, the rest is removed by the MRP1 protein (multidrug resistance protein 1) [34,35,36]. The extent to which both putative mechanisms of MDA reduction are involved deserves separate studies. The initiation of one or another mechanism is most likely associated with a certain threshold level of oxidative stress and the degree of lipid peroxidation in erythrocytes, so the use of GTE does not allow reaching the initiation threshold.

A decrease in GAPD activity indicates a weakening of the glycolytic pathway for glucose utilization, possibly due to oxidative modification of the enzyme, which reduces the generation of NADH and the reducing potential of metHb reductase and other NADH-dependent enzymes. However, judging by the absence of changes in metHb levels, the available amount of NADH is sufficient to restore metHb. Since NADH is formed not only as a result of the activity of GAPD, but also from lactate as a result of the “reverse” activity of LDH (although, in fact, for the erythrocyte isoform LDH2 such activity is “direct”), apparently there is a reorientation of erythrocytes to import lactate in parallel with an adaptive increase in the expression of monocarboxylate transporter type 1 (MCT1) [18]. 1,3-Bisphosphoglycerate formed under the influence of GAPD is apparently utilized predominantly through the Rapoport–Lubering shunt with the formation of 2,3-bisphosphoglycerate (BPG), which is an adaptive reaction of erythrocytes aimed at reducing affinity hemoglobin to oxygen and increasing its release in tissues, as well as maintaining the elasticity (deformability) of red blood cells [37]. Activation of this shunt is associated with a decrease in ATP synthesis in the phosphoglycerate kinase reaction. As a result, Ca^2+^-dependent K^+^-channels are activated; this is associated with the release of water through aquaporins (AQ1), the entry of Cl^−^ ions through the AE1 exchanger and an additional decrease in intracellular pH [38]. The release of water causes a decrease in the volume of erythrocytes and an increase in the concentration of Hb as the main intracellular anion and osmoregulator. Thus, the identified changes in biochemical parameters in the erythrocytes of rats of the GTE + ACL group suggest an increase in the activity of the Rapoport–Lubering shunt and a Ca^2+^-dependent decrease in pH; it is possible to increase ROS-induced eryptosis with subsequent renewal of the erythrocyte pool. Apparently, erythrocytes of this group import lactate not so much to transfer it to slow muscle fibers or the liver, but to generate NADH through LDH and maintain the activity of metHb reductase; this also explains the inverse correlation between lactate levels and the duration of the rats’ swimming on the last day of the experiment. The physiological and biochemical adaptation of erythrocytes in this group of rats is aimed mainly at preserving the active pool, the functional activity of which is steadily decreasing; erythropoiesis may be increased, but its intensity does not correspond to the increased needs of the body.

In animals of the GTE + ACR group, relative to the negative control group, GR activity was reduced by 35% and TMET activity was reduced 3.4-fold, while relative to the NaCl group, total ATPase activity was reduced by 11%. Ammonium preconditioning promotes activation of the main metabolic pathways of erythrocytes, optimization of the processes of binding and release of oxygen, along with the mobilization of the pool of young erythrocytes [29].

Increased energy consumption of erythrocytes must be provided by certain steps of glycolysis that generate ATP; in particular, 3-phosphoglycerate kinase. The Rapoport–Lubering shunt is not activated under these conditions, and the increase in oxygen delivery by erythrocytes is provided primarily by metabolic acidosis as a result of muscle activity under conditions of increased stress associated with the generation of carbon dioxide and pyruvate. Pyruvate is an endogenous LDH inhibitor, which provides an additional decrease in intracellular pH and is effective. implementation of the Bohr effect (pKa of pyruvate is significantly lower than pKa of lactate: 2.45 vs. 3.80). Ammonium salts can serve as a trigger and (or) amplifier of such a mechanism of biochemical adaptation.

The physiological and biochemical adaptation of the erythrocytes of rats taking ammonium salts is apparently aimed at optimizing the oxygen transport function in the conditions of the existing network of blood vessels and their “shuttle” function in relation to lactate. The triggering or enhancing function of ammonium may consist in increasing the activity of carbonic anhydrase and the Cl^−^/HCO_3_−exchanger (AE1) due to an increase in pH_i_, including due to the acceptance of H^+^ near the anion exchanger AE1 by neutral ammonia molecules passing through the RgAG channel [39]. A local increase in intracellular pH reduces the activity of the Rapoport–Lubering shunt [40,41], thus increasing the activity of ATP-generating reactions and causing an increase in the lactate capacity of erythrocytes. High concentrations of carbon dioxide have a stronger effect on the binding of lactate to hemoglobin than high concentrations of lactate on the carbamination of hemoglobin [42], which determines the export of lactate from erythrocytes in the most intensively working slow-twitch muscles, generating a significant amount of carbon dioxide. Available data suggest that the action of ammonium on red blood cells causes an effect opposite to the Root effect, i.e., increasing not only the affinity of oxygen for hemoglobin, but also the cooperativity, capacity and transport ability of hemoglobin [43].

### 2.4. Hematological Parameters of Rats on the Next Day after Final Exercise Load

Eleven hematological parameters were identified out of 16 studied, which did not undergo significant changes in the experimental groups in relation to the negative or positive control: the absolute number of leukocytes—white blood cells (WBCs), the relative number of lymphocytes (Lymph %), absolute and relative number of granulocytes (Gran and Gran %, respectively), red blood cell concentration (RBC), hemoglobin (HGB), hematocrit (HCT %), mean corpuscular volume (MCV), mean concentration hemoglobin (MCH), mean corpuscular hemoglobin concentration (MCHC) and mean platelet volume (MPV) (Table 5).

In rats of the positive control (NaCl) and GTE + ACL groups, no deviations in hematological parameters from the negative control group (Control) were detected. The absolute number of lymphocytes in rats of the GTE group increased relative to the negative control by 38%. Taking into account the lymphocytic profile of rats, this increase seems to be quite significant. Available data from another exercise model in rats suggest improvements in immune function and T- and B-lymphocyte metabolism, although hematological profiles were not the focus of the study [44].

Animals in the ACL group have the maximum number of deviations in hematological parameters, all of which characterize the state of platelets: platelet count (PLT), platelet distribution width (PDW) and the plateletcrit (PCT). All three indicators are significantly different from the indicators of the negative control group: PLT is decreased by 25%, PDW is increased by 3.4%, PCT is decreased by 33%, but PCT is also decreased by 16% in relation to the positive control group. The totality of these data indicates the activation and partial aggregation of platelets of the ACL group, although aggregation does not mean the formation of microthrombi exclusively in the vascular bed, it can already occur in ex vivo conditions in the period between blood sampling and measurements on a hematology analyzer. At the same time, in rats of the ACR group, which demonstrated maximum functional indicators, only a 25% decrease in thrombocrit was detected and only relative to the negative control group, which indicates a slight activation of platelets in rats of this group compared to the ACL group.

Finally, in rats of the GTE + ACR group, with the same median value of thrombocrit, there is no statistical significance of the differences, but the number of platelets was significantly reduced by 18% relative to the negative control. In addition, the RDW was reduced by 11% in rats of this group. A slight decrease in RDW is not associated with any pathology; moreover, it indicates a possible decrease in thrombogenicity, increased generation of nitric oxide, and even increased hematopoiesis [45,46,47].

### 2.5. Immunological Parameters of Rats on the Next Day after Final Exercise Load

Seventeen immunological parameters were identified out of 37 studied, which did not undergo significant changes in the experimental groups in relation to the negative or positive control (not included in the table): the relative number (%) of B-cells, T-cells, and double negative (CD4-CD8-) cells; relative and absolute number of natural killer cells (NK), double positive (CD4+CD8+), central memory T helper cells (Th 44+62L+), mature T helper effector cells (Th 44dim62L−), cytotoxic effector memory T cells (Tcyt 44+62L−); the absolute number of invariant natural killer T cells (NKT), the total number of naive and central memory T helper cells (Th 62+), naive T helper cells (Th 44dim62L+), central memory cytotoxic T cells (Tcyt 44+62L+). It should be noted that indicators in certain groups change: only in relation to the negative control (B-cells, T-cells, Th, CD4-CD8-, Tcyt 62L+, Tcyt 44dim62L+, Th 62L+ %, Th 62L− %, Th 44 +62L− %, Th 44+62L−); only in relation to the positive control (Tcyt, CD4/CD8, Tcyt 62L− %, Tcyt 62L−, Tcyt 44+62L+ %, Tcyt 44dim62L− %, Tcyt 44dim62L−, Tcyt 44dim62L+ %); and in relation to both negative and positive controls (NKT-cells %, Th 44dim62L+ %; the last of the noted immunological parameters is the only one that was significantly changed in one group in relation to both controls) (Table 6).

In the positive control group (NaCl), relative to the negative control group (intact animals), a deviation was detected in only one indicator, NKT-cells %, but quite significant: a decrease of 71%, or 3.4-fold. NKT cells play a critical role in the interface between the innate and adaptive (acquired) immune response, secrete a wide range of cytokines, including gamma interferon (IFN-γ) and interleukin 4 (IL-4), and determine the differentiation of T helper cells 1 and 2 type and provide a primary antitumor response [48,49,50]. Thus, there is some suppression of the immune system in the NaCl group rats.

In the GTE group, 6 indicators of the immunological profile have statistically significant deviations from the negative control group. There is also one Tcyt indicator, which is increased by 68% in relation to the positive control (NaCl group), and indicates the manifestation of an immunoregulatory function in rats of the GTE group against the background of a decrease in this function in rats of the NaCl group. Relative to the negative control, the absolute number of T-cells was increased in the GTE group by 41%; the absolute number of T-helpers is increased by 62%, and the maximum contribution to this change is made by helpers of the 3rd and 4th stages of differentiation (Th 62L−), the number of which is increased by 2-fold; the absolute number of double negative CD4-CD8- increased by 21%; the absolute total number of cytotoxic naive and central memory cells (Tcyt 62L+, 1st and 2nd stages of differentiation) increased by 35%, and this increase was due to a significant increase of only naive Tcyt 44dim62L+ cells by 43%. Thus, in the absence of differences in functional indicators (swimming duration) from the NaCl group, in animals of the GTE group there is stimulation of the immune system without signs of overstrain or failure of adaptation.

In the ACL group, 6 deviations were identified, all in relation to intact animals. The absolute number of B-lymphocytes was increased by 58%; the relative number of NKT-cells was reduced by 68%, or 3.2-fold; the absolute number of Tcyt 62L+ (Tcyt naive and central memory cells, 1st and 2nd stages of differentiation) increased by 49%, mainly due to naive Tcyt 44dim62L+ cells, the number of which was significantly increased by 43%; finally, as in animals of the GTE group, in animals of the ACL group the absolute number of helpers of the 3rd and 4th stages of differentiation (Th 62L−) was increased by 2-fold, mainly due to effector memory T-helpers (Th 44+62L−) the number of which is 2.1-fold higher. Rats of this group demonstrated increased functional results compared to animals of the positive control and GTE groups, however excessive mobilization of the immune system and an emerging imbalance between different clusters of the cells were observed. This may mean a development of inflammatory process and an increase in the specific humoral response.

In the ACR group, only one deviation was revealed, and only in relation to the negative control (intact animals): the relative number of T-helper effector memory cells (Th 44+62L− %) increased by 91%. Thus, in animals that showed maximum functional results, minimal (and at the same time optimal) mobilization of T-cell immunity along the T-helper link is observed without any signs of an imbalance of the immune system.

The GTE + ACL group revealed the only statistically significant deviations from both the negative and the positive control groups. This is the relative number of naive T helper cells (Th 44dim62L+ %), which is reduced by 8% relative to the two control groups. The total relative number of naive T helper cells and central memory cells (Th 62L+ %) was slightly (but statistically significant) reduced by 3% in relation to only the intact control. The total relative number of T-helpers of effector memory and mature effectors (Th 62L− %) was increased in the GTE + ACL group by 47% relative to the negative control, which is due to a significant increase of 94% exclusively in T-helpers of effector memory (Th 44+62L− %). Thus, in animals of the GTE + ACL group, which showed slightly worse functional results compared to animals in the ACL group, moderate mobilization of T-cell immunity due to T-helper cells was revealed.

In the GTE + ACR group, the largest number of significant deviations from the indicators of the two control groups was revealed—fifteen, and at the same time the largest number of reliable deviations—twelve—from the parameters of the positive control group (Table 6 and Discussion section). These 12 parameters comprise: relative number of T helper cells (14% decrease); relative amount of Tcyt (one-and-a-half-fold increase); relative amount of Tcyt of the 1st and 2nd stages of differentiation (62L+ cells, 44% decrease); absolute number of Tcyt cells (56% increase); relative amount of NKT-cells (increase by 4.4-fold); CD4/CD8 ratio (38% decrease); relative and absolute values of the total number of effector memory cells and mature Tcyt effectors (increase by 5.3- and 4.6-fold, respectively); an increase in the level of exclusively mature effectors, Tcyt 44dim62L− % and Tcyt 44dim62L− (5.6- and 4.8-fold, respectively); significant decrease by 41% and 45% in the relative number of cytotoxic naive cells (Tcyt 44dim62L+ %) and central memory cells (Tcyt 44+62L+ %), respectively. The relative number of effector memory and mature effector T-helper cells (Th 62L− %) increased in the GTE + ACR group by 63% relative to the negative control; this change is due to a significant increase in effector memory cells by 97%, i.e., almost twice. At the same time, the expected significant decrease by 4.3% in the relative number of naive and central memory T-helper cells (Th 62L+ %) was not associated with statistically significant changes in separately naive and central memory cells. Thus, the immunological status of animals in the GTE + ACR group, which showed high functional results comparable to the results of rats in the ACR group, differs slightly and not fundamentally from the status of animals in this group. The abundance of significant deviations is mainly due to differences from the indicators of rats in the positive control group in the direction of restoring a potentially suppressed immunity.

### 2.6. Effect of GTE and Low Doses of Ammonium Salts on Gene Expression in Rat Muscles on the Next Day after Final Exercise Load

Before describing the results obtained on gene expression, it should be pointed out that the choice of target genes was determined by the desire to understand the relationship between the mechanisms of calcium regulation and signaling in muscle fibers of different types. Therefore, it would be appropriate to briefly remind the reader of the functions of the proteins encoded by the target genes.

Calcium ATPase (SERCA1 mainly in fast muscles, SERCA2 in slow-twitch skeletal and cardiac muscles) is an ATPase of skeletal muscles, a transporter of calcium from the cytosol to the sarcoplasmic reticulum [51]. Calsequestrin (CASQ1) is a calcium-binding protein within the sarcoplasmic reticulum (SR) acting as a calcium buffer in the SR and as luminal regulator for RYR activity via triadin and junctin [52,53]. The ryanodine receptor of skeletal muscles (RYR1) mediates the release of calcium ions from the sarcoplasmic reticulum, which is a necessary step in muscle contraction [53]. RYR1 is mechanically linked to the dihydropyridine receptor (L-type calcium channel, CACNA1S) in the T-tubule sarcolemma, which opens in response to depolarization (depolarization-induced calcium release, DICR). However, DICR is not strictly determined in skeletal muscles and under certain conditions (tetanus, fatigue, contracture, hypoxia, oxidative stress, etc.) the more typical cardiac muscle mechanism CICR (calcium-induced calcium release) may operate in them [54,55]. PGC-1α is a kind of signaling hub, interacting with nuclear receptors and transcription factors, activating transcription of target genes [56]. Its activity responds to multiple stimuli, including calcium ions, ROS, insulin, thyroid hormones and estrogen, hypoxia, ATP demand and cytokines. It is known that PGC-1α is the main regulator of phenotypic adaptation to physical activity and the transformation of fibers from type 2 to type 1 [57]. Myosin heavy chain genes mediate the phenotype of muscle fibers: type 1 (slow oxidative fibers), 2a and 2xd (fast oxidative fibers), 2b (fast glycolytic fibers) [58]. The phenotype of muscle fibers can change during adaptation to external factors, primarily to physical activity [59].

**Figure 1:**
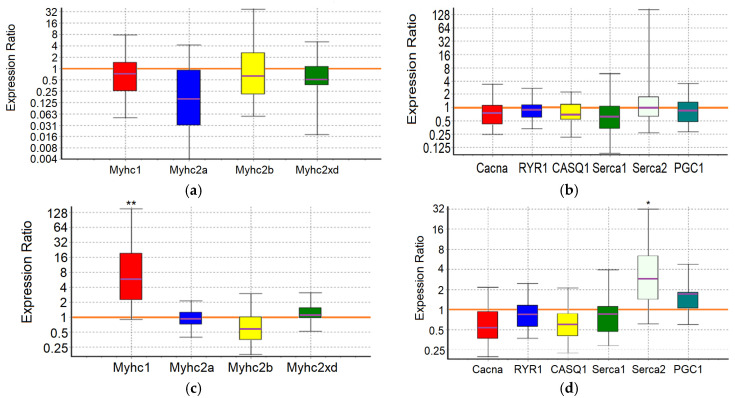
Positive control group (NaCl). Expression of the *Myhc* genes (**a**,**c**), which determine the phenotype of slow-twitch SOL muscles (**a**,**b**) and fast-twitch EDL muscles (**c**,**d**), and genes encoding proteins that regulate the balance of Ca^2+^ ions (**b**,**d**). *, **—differences from the negative control group are statistically significant (*p* < 0.05 and *p* < 0.01, respectively).

An analysis of the expression of 10 target genes in the m. soleus (SOL) and m. extensor digitorum longus (EDL) muscles showed that in the positive control animals, which showed the maximum results in terms of swimming duration in the group, a significant increase in expression was detected in EDL muscles genes *Myhc1* (7-fold, *p* = 0.01) and *Serca2* (3.2-fold, *p* = 0.038) (Figure 1; Table 7 and Table 8).

It is known that in the human body and rodents, almost all relatively fast- and slow-twitch muscles are hybrid, so that a change in the ratio within a particular muscle depends on the nature of the load being performed and is associated with phosphorylation of myosin and a change in sensitivity to Ca^2+^ ions [60,61,62,63,64,65]. During myogenesis, embryonic and fetal myoblast lineages form slow- and fast-twitch primary and secondary myotube ontotypes (myotubes) that respond differently to postnatal neural and thyroid influences, generating fully differentiated fiber phenotypes. Fibers of this phenotype can arise from myotubes of different ontotypes, which retain the ability to respond differently to nervous and thyroid influences during postnatal life [66]. On the other hand, it was estimated that the EDL and SOL of the rat are unusual in being essentially pure fast- and slow-twitch muscles, respectively [67].

Consequently, adaptation to the maximum load in control animals is due to the «transformation» (the balance shift) of the fast-twitch muscles into slow ones, which is associated with the need for enhanced pumping of Ca^2+^ ions from the sarcoplasm. A shift toward type I fiber type was apparent during both endurance and resistance exercise bouts, which elicited increases in intracellular calcium along with the activation of AMPK [65]. The physiological significance of such a restructuring of fast-twitch muscles, instead of their proper specialization, may reside in the emergence of the opportunity to utilize lactate in situ, in conditions of insufficient blood supply and/or insufficient “lactate capacity” of erythrocytes.

**Figure 2:**
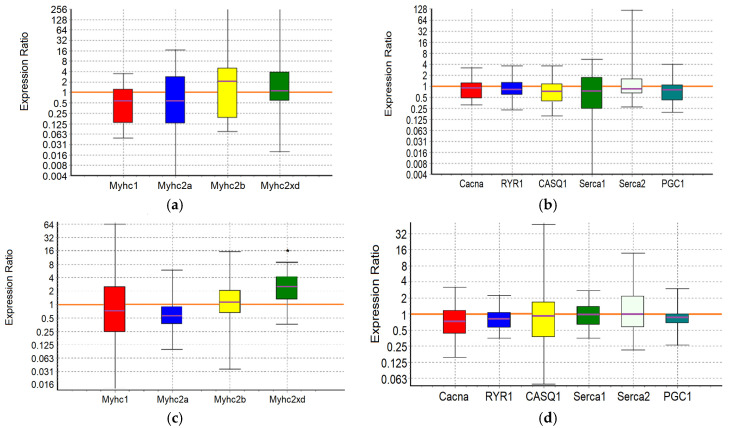
GTE group. Expression of the *Myhc* genes (**a**,**c**), which determine the phenotype of slow-twitch SOL muscles (**a**,**b**) and fast-twitch EDL muscles (**c**,**d**), and genes encoding proteins that regulate the balance of Ca^2+^ ions (**b**,**d**). *—differences from the negative control group are statistically significant (*p* < 0.05).

In the m. soleus of the GTE group, no abnormalities in the expression of the ten studied genes were found (Figure 2; Table 7). In the EDL muscles of the GTE group, a significant increase in *Myhc2xd* expression by 2.2-fold was registered (*p* = 0.045; Table 8). Thus, adaptation to the maximum load in animals of the GTE group is accompanied by an increase in oxidative metabolism solely due to increased expression of *Myhc2xd* in the fast-twitch muscles, with a more even distribution of load between slow- and fast-twitch muscles (compared to the positive control group). This is not supported by signal regulation of PGC1, and does not require the retention of an excess Ca^2+^ ions in the SR (in contrast to, for example, the muscles of animals of the ACR group, see below). The fast type IIx/d muscle fibers are known to be more oxidative as compared with IIb fibers; in addition, their development is known as an “overshoot” phenomenon in which type IIx fibers reach levels above what would be expected with a rest alone after the load [65].

**Figure 3:**
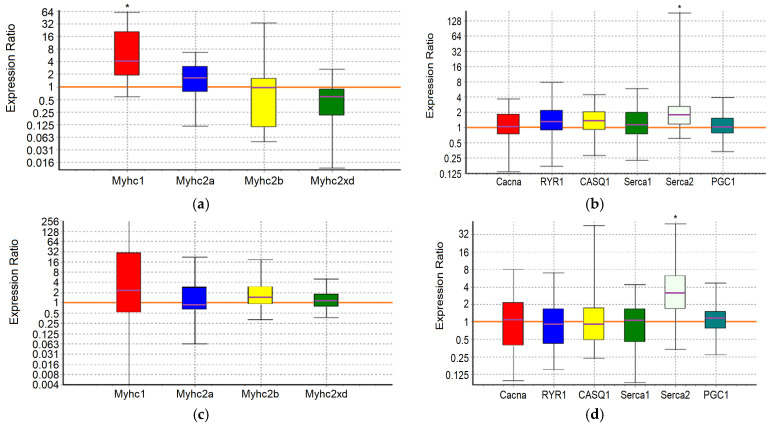
ACL group. Expression of the *Myhc* genes (**a**,**c**), which determine the phenotype of slow-twitch SOL muscles (**a**,**b**) and fast-twitch EDL muscles (**c**,**d**), and genes encoding proteins that regulate the balance of Ca^2+^ ions (**b**,**d**). *—differences from the negative control group are statistically significant (*p* < 0.05).

In the ACL group, the functional effect was due to a significant increase in the expression of the *Myhc1* and *Serca2* genes in SOL by 5.6-fold (*p* = 0.032) and 3-fold (*p* = 0.02), respectively, while in the EDL it was due to an increase in the expression of the *Serca2* gene by 3.4-fold (*p* = 0.042) (Figure 3; Table 7 and Table 8). Thus, ACL contributes to the redistribution of the load from the fast-twitch to slow-twitch muscles, increasing the utilization of oxygen and lactate by the slow-twitch muscles, while the increased pumping of Ca^2+^ ions from the sarcoplasm occurs approximately equally in both types of muscles.

**Figure 4:**
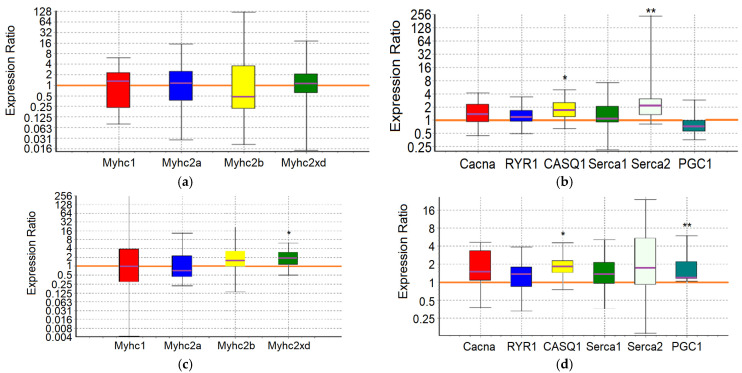
ACR group. Expression of *Myhc* genes (**a**,**c**), which determine the phenotype of slow-twitch SOL muscles (**a**,**b**) and fast-twitch EDL muscles (**c**,**d**), and genes encoding proteins that regulate the balance of Ca^2+^ ions (**b**,**d**). *, **—differences from the negative control group are statistically significant (*p* < 0.05 and *p* < 0.01, respectively).

In the SOL muscles of animals of the ACR group, a significant increase in the expression of *Serca2* gene by 3.6-fold (*p* = 0.004) and *Casq1* gene by 1.7-fold (*p* = 0.036) was revealed. In the EDL muscles of animals of the ACR group, the expression of the *Myhc2xd* gene was increased by 1.8-fold (*p* = 0.028), the *Casq1* gene by 1.8 times (*p* = 0.031) and the *PGC1* gene by 1.6-fold (*p* = 0.003) (Figure 4; Table 7 and Table 8). Thus, adaptation to the maximum load in animals of the ACR group is accompanied by increased pumping of Ca^2+^ ions from the sarcoplasm and their retention in the reticulum of the slow-twitch muscles, while in the fast-twitch muscles, during adaptation, there is an increase in oxidative metabolism due to increased expression of *Myhc2xd* and *PGC1*, along with the increased capacity to retain Ca^2+^ ions in the sarcoplasmic reticulum (SR) and mitochondria. Obviously, in the fast-twitch muscles, the process of mobilization of Ca^2+^ ions from the reticulum is a strictly controlled process, so there is no need for additional energy expenditure by Ca-ATPase to remove Ca^2+^ ions from the sarcoplasm.

**Figure 5:**
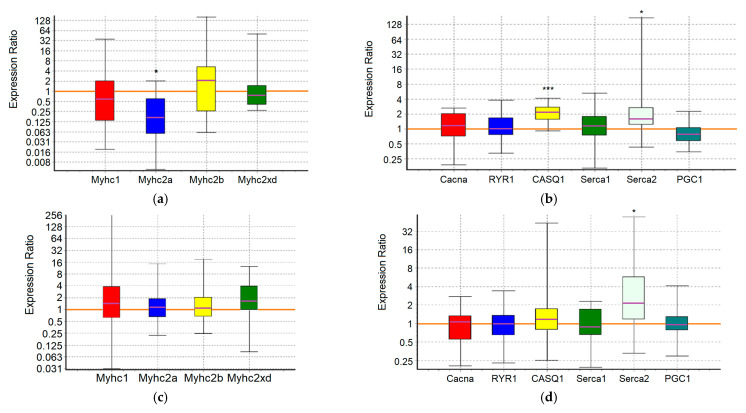
GTE + ACL group. Expression of *Myhc* genes (**a**,**c**), which determine the phenotype of slow-twitch SOL muscles (**a**,**b**) and fast-twitch EDL muscles (**c**,**d**), and genes encoding proteins that regulate the balance of Ca^2+^ ions (**b**,**d**). *, ***—differences from the negative control group are statistically significant (*p* < 0.05 and *p* < 0.001, respectively).

In the slow-twitch SOL muscles of the GTE + ACL group, the expression of the *Myhc2a* gene was significantly reduced by about 6-fold (*p* = 0.047), and the expression of the *Casq1* and *Serca2* genes was increased by 2.1-fold (*p* = 0.001) and 3.3-fold (*p* = 0.032), respectively. In the fast-twitch EDL muscles, a significant increase in *Serca2* gene expression by 2.6-fold (*p* = 0.027) was revealed (Figure 5; Table 7 and Table 8). This may indicate the most economical use of available mitochondria and, in general, involvement of both slow-twitch and fast-twitch muscles because of adaptation. On the other hand, a significant decrease in the expression of the *Myhc2a* gene in slow muscles without a compensatory increase in the expression of other genes of myosin heavy chains, and at the same time an increase in the expression of genes encoding calcium-regulating proteins, may indicate a breakdown of adaptive mechanisms when performing a functional load at a sufficiently high level.

**Figure 6:**
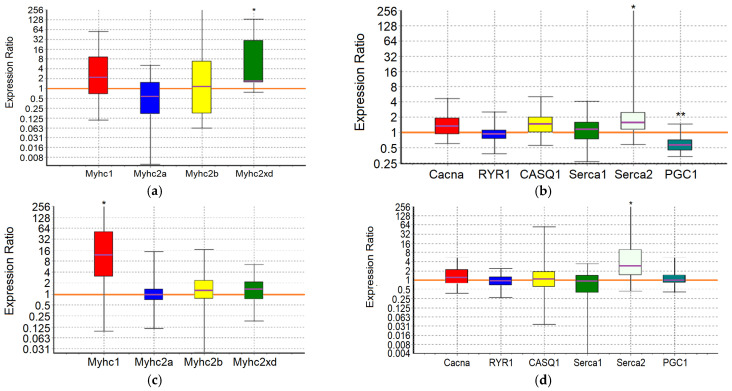
GTE + ACR group. Expression of *Myhc* genes (**a**,**c**), which determine the phenotype of slow-twitch SOL muscles (**a**,**b**) and fast-twitch EDL muscles (**c**,**d**), and genes encoding proteins that regulate the balance of Ca^2+^ ions (**b**,**d**). *, **—differences from the negative control group are statistically significant (*p* < 0.05 and *p* < 0.01, respectively).

In the m. soleus of the GTE + ACR group, a significant increase in *Myhc2xd* expression by 4.3-fold (*p* = 0.035) was registered in combination with an increased expression of the *Serca2* gene by 3.1-fold (*p* = 0.027) and a decrease in the expression of the *PGC1* gene by almost 2-fold (*p* = 0.007). This indicates a kind of “power” adaptation, during which the slow-twitch muscles are transformed into the fast-twitch ones, but remain focused on oxidative metabolism. At the same time, the reverse process is observed in the EDL muscles: the expression of the *Myhc1* gene is increased by more than an order of magnitude (12.3-fold, *p* = 0.012), and the *Serca2* gene by 4.5-fold (*p* = 0.024) (Figure 6; Table 7 and Table 8). It is interesting that in fast-twitch muscles the list of genes “affected” by the load and the direction of changes in their expression coincides with what we observed in the positive control group. However, in the slow-twitch muscles of rats from the GTE + ACR group, a transformation also occurs into the muscles of another type, which indicates the equal involvement of muscle fibers of different types in the process of adaptation to the maximum load.

### 2.7. The Influence of GTE and Small Doses of Ammonium Salts on the Ultrastructure and Morphometric Parameters of Rat Muscles on the Next Day after Final Exercise Load

**Figure 7:**
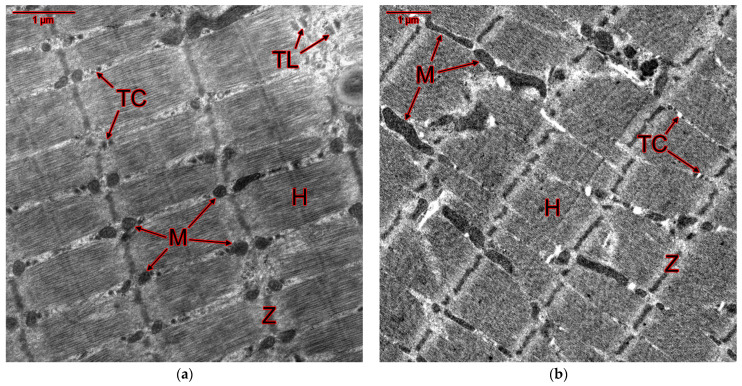

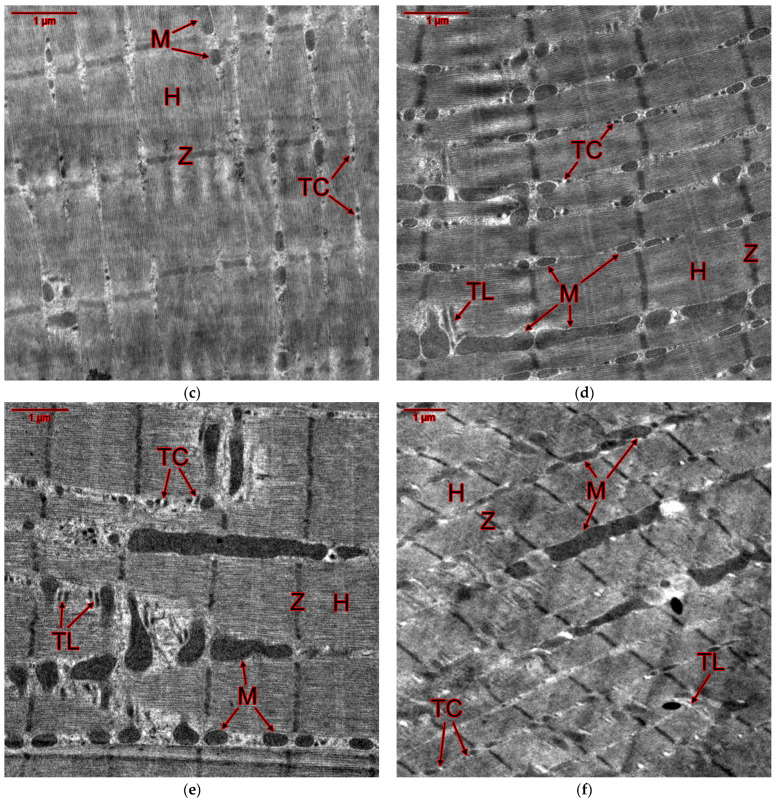

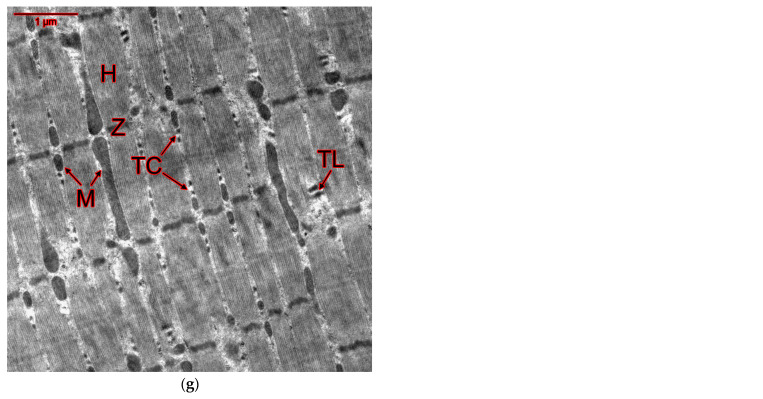
M. soleus of rats: (**a**) negative (intact) control group; (**b**) positive (NaCl) control group; (**c**) GTE group; (**d**) ACL group; (**e**) ACR group; (**f**) GTE + ACL group; (**g**) GTE + ACR group. Abbreviations: M—mitochondria; TC—T-tubules, cross section; TL—T-tubules, longitudinal section; Z—Z-line; H—H-zone.

**Figure 8:**
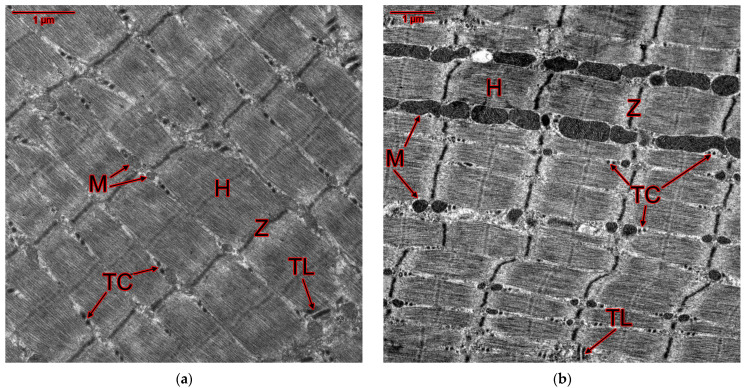

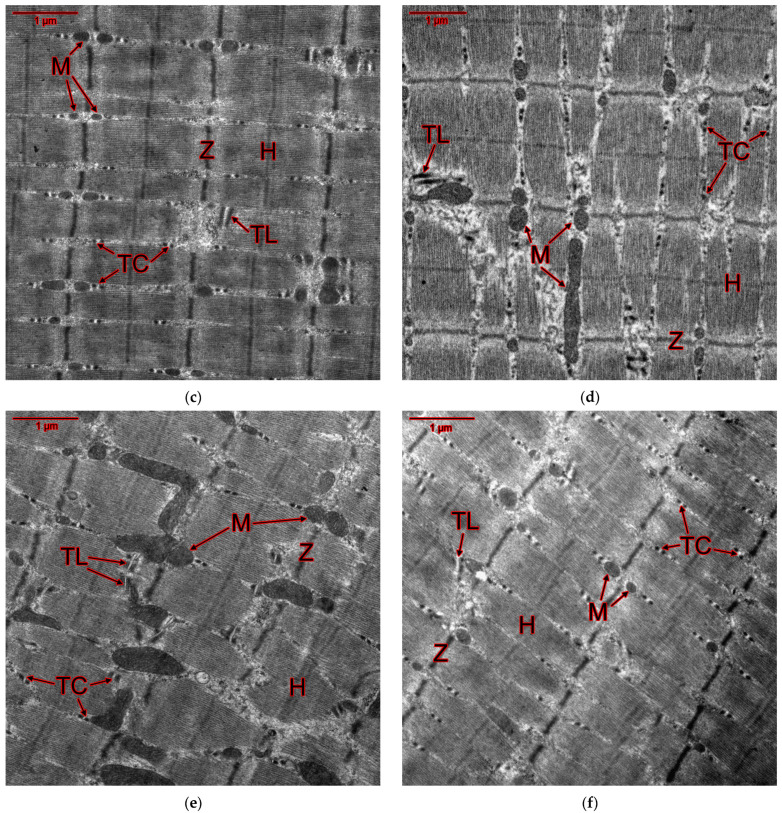

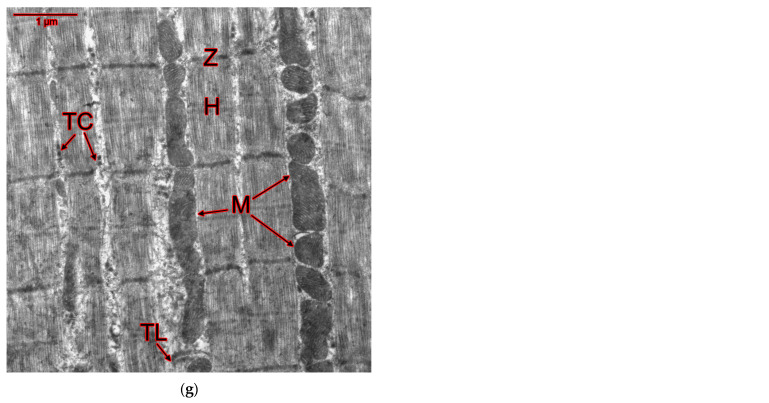
M. extensor digitorum longus of rats: (**a**) negative (intact) control group; (**b**) positive (NaCl) control group; (**c**) GTE group; (**d**) ACL group; (**e**) ACR group; (**f**) GTE + ACL group; (**g**) GTE + ACR group. Abbreviations: M—mitochondria; TC—T-tubules, cross section; TL—T-tubules, longitudinal section; Z—Z-line; H—H-zone.

Electron microscopic and morphometric studies of slow SOL and fast EDL muscles of rats treated with GTE and ammonium salts during a cycle of physical activity, revealed significant changes in the mitochondrial apparatus of rats (Figure 7 and Figure 8; Table 9 and Table 10). First of all, there was an increase in the average mitochondrial area in both slow- and fast-twitch muscles in almost all groups of rats. In addition, changes in the T-tubule system were shown. The differences from the control groups are most pronounced in the ultrastructure of EDL muscle fibers.

In the positive control group, the total lumen of T-tubules in the SOL muscles decreased by 38%, and mitochondrial destruction (cristae deformation and vacuolization) was evident (Figure 7b, Table 9). In EDL muscles, there was a 62% increase in the average mitochondrial area relative to the negative control group, and mitochondrial enlargement and fusion occurred (Figure 8b; Table 10).

In rats of the GTE group, a 22% decrease in the average lumen area of T-tubules was detected in the SOL muscles relative to the intact control (Figure 7c, Table 9). In addition, the average area of mitochondria in the EDL muscles was reduced relative to the positive control by 47% (Figure 8c; Table 10), i.e., almost 2-fold, not significantly different from the indicators of the intact control. Consequently, in animals of the GTE group, the slow-twitch muscles are weakly involved in the adaptation process, but in the fast-twitch muscles, unlike the positive control group, adaptation occurs not due to an increase in the proportion of slow-twitch fibers with an abundance of mitochondria, but due to an increase in the proportion of fast-twitch fibers of the oxidative type, as evidenced by changes in *Myhc2xd* gene expression (Figure 2c).

In rats of the ACL group, the average mitochondrial area increased relative to the intact control by 19% in SOL muscle fibers and 1.9-fold in EDL muscle fibers (Figure 7d and Figure 8d; Table 9 and Table 10). In SOL muscles, the lumen area of T-tubules decreased by 28% relative to the negative control. At the same time, the area of longitudinally cut T-tubules in EDL muscle fibers increased by 47%. According to the data obtained, in slow- and fast-twitch muscles the load is redistributed in favor of slow ones with high oxidative metabolism, which ensured high functional indicators. This in turn caused damage to fast-twitch muscle fibers, in which an almost 3-fold increase in the expression of the *Myhc1* gene did not reach a statistically significant level (Figure 3c).

In rats of the ACR group, the average mitochondrial area increased by 21% in SOL muscles and by 1.9-fold in EDL muscles relative to the intact control, as in rats of the ACL group (Figure 7e and Figure 8e; Table 9 and Table 10). The lumen area of T-tubules in the fast-twitch muscles of rats of this group alone was increased by 1.5-fold relative to the negative control and 2-fold relative to the positive control. However, it was reduced by 24% relative to intact control in the slow-twitch muscles. Also, in rats of this group, the area of longitudinally cut T-tubules increased both in the EDL muscles (by 78% relative to the negative and by 40% relative to the positive control) and in the SOL muscles (by 40% relative to the intact control). According to the data obtained, in the slow- and fast-twitch muscles the load is relatively evenly distributed between slow- and fast-twitch muscles with high oxidative metabolism. This ensured the highest possible functional performance, which in turn caused damage to both fast- and slow-twitch muscle fibers.

In rats of the GTE + ACL group, the area of mitochondria in slow-twitch muscles was increased by 2-fold relative to intact animals (Figure 7f; Table 9). However, along with the enlargement (fusion) of mitochondria, their vacuolization and dismemberment (fission) occurs. In fast-twitch muscles, the area of mitochondria was reduced relative to the positive control by 1.6-fold (Figure 8f; Table 10). T-tubule lumen increases in SOL muscles by 43% and in EDL muscles by 57% relative to the positive control. It should be especially noted that only in animals of this group the cross-sectional area of T-tubules was not reduced relative to the negative control. In both slow- and fast-twitch muscle fibers of rats of this group, the area of longitudinally cut T-tubules increased (2- and 1.6-fold, respectively) relative to intact animals. Also, this area is increased in both slow- and fast-twitch muscles by 1.7- and 1.2-fold, respectively, relative to the NaCl group. Thus, in rats of the GTE + ACL group, adaptation of muscle fibers is ensured mainly by SOL muscles, in which, along with a developed mitochondrial apparatus, there are signs of adaptive restructuring of the EMC system.

In animals of the GTE + ACR group, the largest increase in the average area of mitochondria in EDL muscle fibers was observed among all experimental groups—2.1-fold (Figure 8g; Table 10), which is associated with a maximum increase in *Myhc1* gene expression in fibers of this type (Figure 6c). The mitochondrial area, although not as significantly, also increased relative to the positive control (by 27%). In slow-twitch muscles, the mitochondrial area also slightly increased (1.2-fold), but only in relation to the negative control (Figure 7g; Table 9). In the SOL muscles of rats of this group, the lumen area of T-tubules was reduced by 24% relative to the intact control. The lumen area of T-tubules in the EDL muscles of this group increased by 57% relative to the positive control (Figure 8g; Table 10). The average area of longitudinally cut T-tubules in the EDL muscle fibers increased by 77% relative to the negative control and by 39% relative to the positive control. The data obtained indicate an increased load on the fast-twitch muscles in rats of the GTE + ACR group, and during the adaptation process there is an increased “transformation” of fast-twitch muscles into slow ones, which is accompanied by a powerful strengthening of the mitochondrial apparatus and the EMC system, ultimately—an increase in functional adaptation.

## 3. Discussion

Prior studies have found that the administration of high levels of ACL (0.3 g/kg) has a negative effect on endurance. For example, Hollidge-Horvat et al. [68] describes the administration of ACL in order to induce metabolic acidosis. In the prominent work of Carr et al. [69], a meta-analysis was presented of the effects of acute ingestion of three agents—sodium bicarbonate (SB), sodium citrate (SC) and ACL—on performance and related physiological variables (blood bicarbonate, pH and lactate). It was found that SB produced a “possibly moderate” performance enhancement of 1.7% with a typical dose of ~0.3 g/kg in a single 1-min sprint; SC with a typical dose of ~0.5 g/kg had an unclear effect on performance of 0.0%; and finally, ACL produced a “moderate performance impairment” of 1.6% with a typical dose of ~0.3 g/kg. In [70], the recovery kinetics of pH and lactate for the conditions of pre-exercise acidosis, alkalosis and placebo states was measured. Twelve trained male cyclists completed three exercise trials (110% workload at VO2max), ingesting either 0.3 g/kg of ACL, 0.2 g/kg of NaHCO_3_ and 0.2 g/kg of sodium citrate, or a placebo (calcium carbonate). Blood samples were drawn before, during, and after exercise. Exercise-induced acidosis was more severe in the ACL and placebo trials.

In our previous works, we have found that, surprisingly, the administration of low levels of ammonium salts can actually enhance endurance [21,22,23]. The simplest explanation that comes to mind when comparing the conflicting results obtained in experiments on humans and on rats (or other animals) is that animal models do not always provide results transferable to what occurs on human subjects, and vice versa. However, we emphasize once again that we are talking first and foremost about the use of doses that differ by more than an order of magnitude, as well as about the mode of administration of different doses of ammonium salts. Without denying the need to make adjustments for species differences, the presence of common biochemical and physiological mechanisms of action of ammonium salts has been proven in experiments with human volunteers, in which low-dose ACL enhanced endurance in submaximal physical exercises [24].

Enhancement of physical endurance may be achieved by delaying onset and/or reducing muscle fatigue. We suggested that ammonium salts have a pleiotropic effect, which includes: (1) transformation of the fast-twitch muscles; (2) enhancement of expression of Ca^2+^ regulators, such as Ca-ATPases, calsequestrin, etc.; (3) enhancement of expression of metabolic regulators, such as PGC-1a, AMPK, etc.; (4) enhancement of expression of metabolic and antioxidant enzymes, such as LDH1/2, PEPCK, superoxide dismutase, etc.; (5) improvement of oxygen delivery by red blood cells; (6) elevation of the threshold of pain perception by the central nervous system; (7) acceleration of recovery after daily physical load. As a result, muscle fatigue is reduced and the onset of muscle fatigue is delayed. The administration of ammonium salts may result in a reduction in the amount of lactate that is generated in the body and/or higher utilization of lactate in the liver, erythrocytes and muscles.

In the forced swimming model that we had developed, we used rats divided into seven groups and, at the end of the cycle, we identified and compared between the groups a large number of indicators, which were divided into seven groups: functional (swimming duration); blood plasma biochemistry; erythrocyte biochemistry; hematology; immunology; gene expression of slow- and fast-twitch muscle fibers; and ultrastructure and morphometric parameters of slow- and fast-twitch muscle fibers. The dynamics of changes in all groups of indicators in all groups of animals relative to negative and positive controls are given in Table 11 and Table 12, respectively.

Regarding the negative control group, in all groups of animals, unidirectional statistically significant changes were detected in only one indicator, i.e., lactate level 5 min after the final load (Table 11). In five out of six groups, a decrease in the average cross-sectional area of T-tubules in slow-twitch muscle fibers was noted. The exception was the GTE + ACL group, for which the average area of the longitudinal section of T-tubules was maximally increased. In four groups out of six, an increase in the expression of the *Serca2* gene was detected in both slow and fast muscle fibers, but with some differences between the groups: in SOL muscles, *Serca2* expression was increased in the ACL, ACR, GTE + ACL, GTE + ACR groups; in EDL muscles, *Serca2* expression is increased in the NaCl, ACL, GTE + ACL, and GTE + ACR groups. For the GTE group there was no changes in the expression of this gene at all, whereas for the ACL, GTE + ACL, GTE + ACR groups, an increase in the expression of Serca2 was noted in both types of muscle fibers. In rats of the ACR group, we note a connection between the absence of changes in the expression of *Serca2* in the EDL muscles and a significant increase in the expression of *Myh2xd*, *CASQ1* and *PGC1*, as well as an increase in the average cross-sectional area of T-tubules only in rats of this group. Probably, an increase (strengthening) of EMC determines the controlled and well-regulated mobilization of Ca^2+^ ions, which is facilitated by the retention of these ions by calsequestrin and mitochondria. Therefore, there is no need for increased work of Ca-ATPase, additional production of this enzyme and additional energy costs. At the same time, the increased level of Ca^2+^ ions causes an increase in the expression of *PGC1* and the associated generation of mitochondria for renewed fast muscle fibers (increased expression of *Myh2xd*). CREB (cAMP response element-binding protein) and TORCs (transducers of regulated CREB-binding proteins) strongly induce the PGC-1α signaling pathway, linking external signals to the transcriptional program of cellular events. In muscle cells, calcium-signaling components modulate the expression of PGC-1α, in which CREB is a key player [71].

In four out of six groups, an increase was detected relative to the negative control in two ultrastructural parameters of muscle fibers—the average area of mitochondria and the average area of the longitudinal section of T-tubules. Changes in mitochondria in SOL muscles were noted in the ACL, ACR, GTE + ACL, GTE + ACR groups; in EDL muscles—in the NaCl, ACL, ACR, GTE + ACR groups. Animals of the GTE + ACL group according to this trait are clearly in a state of maladaptation: in the EDL muscles there is not even a tendency to increase the mitochondrial area, while in the SOL muscles the giant fused mitochondria undergo vacuolization and fission due to overload. The average longitudinal section area of T-tubules in fast EDL muscles was increased in the ACL, ACR, GTE + ACL, and GTE + ACR groups, while in SOL muscles it was increased only in two groups, ACR and GTE + ACL. It should be noted that only in the ACR group in the EDL muscles the average cross-sectional area of T-tubules was increased, which indicates an increase in the EMC apparatus in rats of this group, which showed maximum functional results on average after 4 days of maximum load.

Regarding the negative control, the maximum number of changes (twenty-two) in all groups of indicators was recorded in the GTE + ACR group, the animals of which showed the maximum functional result and minimum lactate values 5 min and 1 h after the end of the maximum load on the last day of the experiment. Next in terms of the number of changes are the groups ACR (20), ACL (18), GTE + ACL (17), GTE (10) and NaCl (8). In general, the number of identified adaptive changes is directly proportional to the functional state of the animals of the corresponding groups, in terms of the duration of the swimming load in the last 4 days of the experiment. However, not only the quantity, but also the qualitative composition of the identified changes is important. The maximum quantitative contribution to the overall spectrum of changes in the GTE + ACR group (with the maximum number of adaptive changes) is made by biochemical indicators indicating an increase in aerobic lipid utilization, oxidative stress and inflammatory processes (decrease in creatinine and transferrin), a decrease in the level of anabolic processes (decrease in creatinine, HDL, cholesterol) [72,73,74,75]. A decrease in transferrin can lead to iron deficiency in the composition of iron-sulfur complexes in skeletal muscles: in rats of this group, a maximum increase in the area of mitochondria in fast-twitch muscle fibers and, although not the maximum, but a statistically significant increase in slow-twitch fibers, were detected. In addition, physical exercise produces a decrease in erythrocyte concentrations of Fe, Mg and P; this situation could cause alterations in the performance of athletes given the importance of these elements [76].

A decrease in the number of platelets, as well as indices of RDW, GR and TMET in erythrocytes indicates, firstly, the reciprocal relationships of these cells in the hematopoietic system [77]; secondly, about the high functional activity of the existing pool of erythrocytes and the increased level of oxidative stress in these cells [78,79,80].

There are at least two reciprocal NADH-dependent enzymatic mechanisms in erythrocytes: NADH-quinone reductase (EC 1.6.5.9), which is part of the erythrocyte antioxidant system, mediating transmembrane electron transfer, and cytochrome-b5 reductase (CYB5R, EC 1.6.2.2), which ensures the reduction of metHb [81,82,83,84]. The latter enzyme has different isoforms, with highly conserved catalytic domain [85]. The presence of a soluble isoform CYB5R3 was described in erythrocytes as the main responsible for the enzymatic recycling of metHb [82,86,87,88]. CYB5R3 has been shown to function without CYB5, as shown with electron transfer to CoQ to stabilize ascorbate [89]. Under stress conditions, CYB5R3 maintains membrane embedded α-tocopherol and ascorbate, potent membrane antioxidants in living cells, in their reduced state [84]. Obviously, the decrease in TMET in the erythrocytes of rats of the ACR and GTE + ACR groups revealed in our experiments is due to the close coupling of the soluble form of CYB5R3 with hemoglobin, which allows metHb to be restored with a minimal lag period as soon as it is formed, preventing its accumulation.

In both types of muscles of GTE + ACR group rats, the expression of the Ca-ATPase gene (*Serca2*) is increased, but a specific feature is an increase in the expression of the slow fiber gene in fast-twitch muscles and the fast oxidative fiber gene in slow-twitch muscles, along with a decrease in the expression of the *PGC1* gene in them. On one hand, it is a well-known phenomenon that reduced neuromuscular activity, hyperthyroidism or mechanical unloading stimulate slow-to-fast fiber type transitions, while increased neuromuscular activity, hypothyroidism and higher mechanical loading result in fast to slow fiber type transitions [90]. On the other hand, in the SOL muscle mRNA expression of slow *Myhc1* remained up to three orders higher compared to fast *Myhc* transcripts, which explains the predominance of MyHC-1 isoform and fiber type 1 even in hyperthyroid rats [91]. The EDL and SOL of the rat are unusual in being essentially pure fast and slow muscles, respectively [67,92]. Although hyperthyroid status led in the SOL to increased expression of *Myhc2a* mRNA, MyHC-2a isoform and 2A fibers, it preserved extremely low expression of *Myhc2x* and *-2b* mRNA and protein isoforms, which explains the absence of pure 2X/D and 2B fibers. Hypothyroid status, on the other hand, almost completely abolished expression of all three fast MyHC mRNAs, MyHC protein isoforms and fast fiber types in the SOL muscle [75]. Experimental denervation induced skeletal muscle atrophy, mitophagy and slow-to-fast muscle fiber type transition [67]. Reprogramming of fast-to-slow myofiber switch can improve endurance capacity and alleviate fatigue [93]. Nevertheless, it obviously does not explain what we have revealed in our experiments. We suggest that such a radical molecular genetic restructuring indicates an overload of fast and slow muscles in rats of this group. This is also evidenced by a significant increase in the average area of the longitudinal section of T-tubules in the EDL muscles, while in the SOL muscles the average area of the transverse section of T-tubules is reduced. Immunological changes due to a significant increase in the relative number of peripheral memory T helper cells (third stage of differentiation)—with a simultaneous significant decrease in the proportion of naive helper cells and central memory cells—indicate a high degree of mobilization of the immune system.

In rats of the ACR group, which showed the maximum average swimming time and almost equal duration of swimming on the last day of the experiment with the rats of the GTE + ACR group, the maximum quantitative contribution to the spectrum of identified changes—11 out of 20—is made by structural (morphometric) and molecular genetic indicators. The average mitochondrial area was increased in slow and especially fast-twitch muscle fibers; similarly, to a greater extent in fast fibers, the average area of the longitudinal section of T-tubules was increased. At the same time, the average cross-sectional area of T-tubules was reduced in the SOL muscles, but significantly increased in the EDL muscles, and this increase was detected exclusively in rats of this group. It characterizes increased coupling of the EMC system and correlates with an increase in the expression of *Myh2xd*, *CASQ1* and *PGC1a* genes in EDL muscles. In SOL muscles, no significant changes in the expression of myosin heavy chain genes were detected, but adaptation is facilitated by increased expression of the calsequestrin and Ca-ATPase genes. A decrease in the concentration of lactate in both blood plasma and erythrocytes was noted only in rats of this group, which indicates increased utilization of lactate by slow-twitch and/or oxidative fast-twitch muscles and erythrocytes during exercise and/or by the liver for the needs of gluconeogenesis after the end of the exercise. There are three pathways of lactate transport (band 3 system, nonionic diffusion, and monocarboxylate pathway) into erythrocytes [94]. Blood lactate disappearance was faster in trained than untrained subjects during combined active recovery [95].

A decrease in the level of urea and uric acid may indicate a decrease in purine catabolism and the associated work of metabolic pathways in vascular endothelial cells, which are an independent source of these metabolites [96,97]. Increased load leads to moderate mobilization of the immune system, solely due to an increase in the relative number of effector memory helpers.

The range of biochemical parameters of blood plasma and erythrocytes, changes in which were detected one day after the end of the forced swimming cycle, suggests that the differences in the functional state of rats in the ACR and GTE + ACR groups are due to increased carbohydrate metabolism in the ACR group and lipid metabolism in the group GTE + ACR. In the latter case, adaptation is associated with the risk of hepatotoxicity, the causes of which may be different. It should be noted that GTE itself has a hepatotoxic effect in doses significantly (about an order of magnitude) higher than the daily therapeutic dose we use [98]. At the same time, GTE, even in therapeutic doses, can exhibit or enhance the hepatotoxic effect of other substances [99].

In connection with the peculiarities of the action of GTE, the decrease in GR activity in the erythrocytes of rats of the ACR, GTE + ACR and GTE + ACL groups should be discussed. GR is known to be inhibited by some flavanoids, and the following order of potency for inhibition of GR was determined: anthocyanidin > dihydroflavonol = chalcone > flavonol > catechin [100]. It is clear that GTE catechins cannot be considered the reason for the decrease in GR activity in the GTE + ACR and GTE + ACL groups (see below), primarily because a decrease in GR activity was also noted in animals of the ACR group. In addition, in our experiment, the doses of catechins used in the composition of ECG are clearly insufficient to provide an inhibitory effect. Therefore, the explanation for the decrease in GR activity is obviously associated with high physical activity, which is associated with the redistribution and increased need of the body for flavin nucleotides—cofactors of many oxidoreductases [101]. Riboflavin is known to protect tissue from oxidative damage, and riboflavin supplementation before and during prolonged running might reduce muscle pain and soreness during and at the completion of the exercise and may enhance early functional recovery after the exercise [102]. Importantly, erythrocyte glutathione reductase activation coefficient is a measure of riboflavin tissue saturation [103]. On the other hand, reduced erythrocyte GR activity in physically active old men and women as compared to young and physically inactive older people is regarded as an adaptive mechanism which can shift the balance of glucose metabolism in favor of glycolysis especially when catalase activity is not changed [104].

A similar, but more pronounced difference in functional results was found between rats of the ACL and GTE + ACL groups. That is, GTE generally weakens the functional status of rats achieved by using ACL. In rats of the ACL group there are no changes in biochemical blood parameters one day after the end of the load; the main number of changes are distributed between hematological, immunological, morphometric and molecular genetic parameters. The load falls to a greater extent on the fast-twitch muscles, because in them, the average area of mitochondria and the average area of the longitudinal section of T-tubules are significantly increased, whereas in slow-twitch muscles, a less pronounced increase in the average area of mitochondria is associated with a decrease in the average area of the cross-section of T-tubules. At the same time, only in this group of rats the expression of the *Myhc1* gene was increased in the slow-twitch muscles and the expression of the Ca-ATPase gene was increased in both types of muscle fibers. Among the immunological indicators, an increase in the absolute number of B cells (only in this group of rats) and naive cytotoxic T cells, along with an increase in T helper effector memory cells, should be noted. These changes indicate a significant stimulation of humoral and cellular immunity in rats of the ACL group, compared with which in rats of the GTE + ACL group there was an increase only in the relative number of T-helper effector memory cells due to a decrease in the proportion (but not the absolute number) of naive cells.

The average area of mitochondria in rats of the GTE + ACL group increased only in slow muscles, while the average area of the longitudinal section of T-tubules increased in both types of muscles. Of the biochemical parameters of blood plasma one day after the final load, only a decrease in creatinine was noted, which indirectly indicates a weakening of protein metabolism (a similar decrease in creatinine was detected in rats of the NaCl and GTE + ACR groups) and is a surrogate marker of sarcopenia [105]. Only in rats of the GTE + ACL group was the GAPD activity significantly reduced and the BPG level increased, which, along with a decrease in GR activity and lactate levels in erythrocytes, indicates activation of the Rapoport–Lubering shunt and pentose phosphate pathway in erythrocytes during their adaptation to the maximum oxygen delivery to skeletal muscles during physical activity. This conclusion is confirmed by data from a comparative analysis with the positive control group (Table 12). It was revealed long ago that GAPD can form complexes with enzymes which use 1,3-diphosphoglycerate as substrate, namely 3-phosphoglycerate kinase (PGK) at pH 8.0 or 2,3-diphosphoglycerate mutase (DPGM) at pH 6.5 [40]. Later it was shown that a mild oxidation of GAPD must result in acceleration of glycolysis and in decrease in the level of 2,3-diphosphoglycerate due to the acyl phosphatase activity of the mildly oxidized enzyme [106]. GAPD is easily oxidized under oxidative stress, and numerous oxidative modifications of GAPD lead to its denaturation and aggregation, with catalytic Cys152 being important for these processes [107]. Based on these data, we conclude that prolonged oxidative stress in erythrocytes caused inhibition of GAPD, that was bound with PGK at alkaline pH, then the rate of glycolysis was inhibited and accumulation of BPG took place in erythrocytes of the GTE + ACL group. In addition to its compensatory function, the Rapoport–Lubering cycle probably also plays a role in regulating the mass and energy balance of glycolysis, in particular providing increased formation of NADH without a subsequent increase in ATP concentration in respiratory alkalosis [108,109].

In Section 2.3 we considered the paradoxical decrease of MDA level for the ACL group compared to both controls, taking into account that this effect did not occur in co-supplementations with GTE. Since the significant decrease in the median is due to a striking decrease in the upper limit and in the range of MDA values in the ACL group compared to other groups, we suppose either an increased renewal of the erythrocyte pool, or the mechanism for removing MDA from cells was involved, or there was a combination of both mechanisms. It could be suggested that the initiation of one or another mechanism is associated with a certain threshold level of oxidative stress and the degree of lipid peroxidation in erythrocytes, so the use of GTE does not allow reaching the initiation threshold. Ultimately, initiation may be the result of phase changes in a number of parameters, when their interference causes additivity or synergism effects.

The GTE + ACL group is the only one whose representatives do not have increased expression of myosin heavy chain genes in both fast and slow muscles. The adaptive decrease in protein-synthesizing function is confirmed by a decrease in the expression of the *Myhc2a* gene with a simultaneous increase in the expression of the *CASQ1* gene in slow fibers, as well as an increase in the expression of Ca-ATPase genes in both types of muscles. This is associated with destructive changes in tissues, as evidenced by vacuolization and destruction of mitochondria (mitoptosis) in SOL muscles and an increase in the average area of the longitudinal section of T-tubules in both types of muscles in rats of the GTE + ACL group. According to Soukup & Diallo [91], hyperthyroid status led in the SOL to increased expression of *Myhc2a* mRNA, isoform and 2A fibers, at the expense of *Myhc2x* and -*2b* mRNAs and corresponding protein isoforms. On the other hand, hypothyroid status abolished expression of all three fast *Myhc* mRNAs, protein isoforms and fast fiber types in the SOL muscle [91]. It would be interesting to study the hormonal profile of rats upon these experimental conditions.

In the group of GTE rats, which showed no differences in swimming duration from the positive control group, the main changes were due to a block of hematological and immunological parameters. Only in this group of rats the level of lymphocytes, T cells and T helper cells was significantly increased, mainly due to Th62L- effector cells. The absolute number of cytotoxic T-cells is also increased, but at the expense of naïve Tcyt44dim62L+ cells. Moreover, only in GTE group rats the level of double negative cells, which perform an important immunomodulatory function, is increased, along with double positive cells, NKT, Tregs and some other subpopulations of immune system cells [110]. Subpopulations of TCRαβ+ and TCRγδ+ double negative cells perform largely antagonistic functions in inflammation processes, so it is interesting to note that arachidonic (cis,cis,cis,cis-5,8,11,14-eicosatetraenoic, 20:4(n-6)) and adrenic (cis,cis,cis,cis-7,10,13,16-docosatetraenoic 22:4(n-6)) acids induced apoptosis of TCRαβ+ DNT cells and decreased their immunosuppressive function, which were mainly associated with the AKT signaling pathway during nonalcoholic fatty liver disease development [111]. Arachidonic acid also facilitated IL17A secretion by TCRγδ^+^ DNT cells, which was mainly associated with the NF-κB signaling pathway. Therefore, the increase in lipid metabolism in the GTE group may contribute to a shift in the balance towards the suppression of inflammatory functions. To confirm this assumption, it is necessary to determine the metabolomics profile of esterified and non-esterified fatty acids. It should also be noted here that there was a significant decrease in the relative level of NKT-cells in the positive control group, which was not observed in the GTE group and other groups of animals. That is, GTE does not increase the physical performance of rats, but promotes the complex mobilization of immunity with enhanced immunomodulatory function.

The GTE group is an experimental group of animals for which, as in the positive control group (NaCl), no changes in gene expression were registered in the slow-twitch muscles, as compared to the negative controls. In the EDL muscles of animals of the GTE group, a significant increase in the expression of only one of the studied genes, *Myhc2xd*, was noted. A significant increase in the expression of the “housekeeping” gene *Myh2xd* in fast-twitch EDL fibers may be responsible for exercise performance without visible ultrastructural changes in these muscles, while in slow-twitch SOL muscles the average cross-sectional area of T-tubules decreases.

Our data are confirmed by data from other researchers. The suppressive effect of extreme stress on the immune system and the positive immunomodulatory effect of polyphenols have been noted in a number of studies in recent years, both in humans and in animals [112,113,114]

In rats of the positive control group with a minimal number of adaptive changes in the studied parameters in the fast-twitch EDL muscles, the expression of the slow-twitch muscle fiber genes *Myhc1* and Ca-ATPase significantly increases: *Myhc1* by 7-fold and *Serca2* by 3.2-fold. Together with the trend towards a decrease in *Myhc2a* expression in SOL muscles, this indicates that the main load in control animals falls on the fast-twitch muscle fibers. Lactate formed in fast-twitch fibers probably does not go beyond these muscles and must be utilized in situ by increasing the proportion of slow-twitch fibers and increased pumping of Ca^2+^ ions from the sarcoplasm of fast-twitch fibers. At the same time, in the slow-twitch muscles of control animals, there is a tendency for a decrease in the proportion of fast-twitch fibers of oxidative type. In fast-twitch muscles, the average area of mitochondria increases, some mitochondria have a cleared, sometimes blurred matrix, giant mitochondria are found, vacuolization and homogenization of cristae are noted—signs indicating the development of mitoptosis. In slow-twitch muscles, the average cross-sectional area of T-tubules decreases, which indicates functional inhibition of the EMC apparatus. Adaptation to increased load actually does not occur.

In addition, in the positive control group, blood lactate was maximally increased 5 min after the end of the final load, and in the erythrocytes of rats only in this group the level of pyruvate was reduced, which—in the absence of significant changes in the level of BPG—indicates primarily a decrease in the level glycolysis in erythrocytes, but also about the absence of significant transport of exogenous lactate into erythrocytes by any of three pathways of lactate transport (band 3 system, nonionic diffusion, and monocarboxylate pathway) [94,95,115]. Conversion of exogenous lactate to pyruvate via LDH to form reduced NADH is necessary for metHb reduction [116].

As noted above, in relation to the positive control, in the rats of the GTE group there was no increase in the duration of swimming either on the last day of the load or on average over 4 days of maximum load. Also, no changes in the biochemical parameters of erythrocytes and blood plasma were detected one day after the completion of the last load (Table 12). Only an increase in the absolute number of cytotoxic lymphocytes and a decrease in the average area of mitochondria in fast-twitch EDL fibers were registered. The latter circumstance can be explained by the already mentioned increase in the expression of the *Myh2xd* gene and the associated renewal of muscle fibers. These differences from positive control rats indicate a more even distribution of the load between slow- and fast-twitch muscles and accelerated recovery of both types of muscle fibers after exercise.

The animals of the ACL and ACR groups each had five significant deviations from the positive control group, despite the fact that the rats of the ACR group showed more pronounced functional results. Biochemical parameters of erythrocytes in both groups are characterized by an increase in antioxidant status, more pronounced in rats of the ACL group. Morphometric indicators are distinguished by a significant increase in the average area of longitudinal and especially transverse sections of T-tubules in rats of the ACR group, which emphasizes the balanced nature of adaptation mechanisms with maximum functional indicators.

In rats of the GTE + ACL and GTE + ACR groups, the number of significant differences from positive control rats is 2- and 3-fold greater, respectively, than in rats of the ACL and ACR groups. At the same time, only the GTE + ACR group had functional indicators comparable to the ACR group. Biochemical parameters of erythrocytes in the GTE + ACL group, as well as in the ACL group, indicate an increased antioxidant status and activity of the pentose phosphate pathway, while in rats of the GTE + ACR group, a decrease in ATPase activity indicates a decrease in the amount of these proteins as a result of increased glycolytic activity and ATP levels. When compared with the positive control group, the differences between the GTE + ACL and GTE + ACR groups in immunological parameters are especially striking. Thus, in rats of the GTE + ACL group, only a decrease in the relative number of naive T-helper cells was detected, while in rats of the GTE + ACR group, changes were detected in almost all fractions of cytotoxic T-lymphocytes towards their general increase and a shift in the balance to the right—in favor of more differentiated effector cells. Morphometric indicators indicate more pronounced adaptive changes in both types of muscle fibers in rats of the GTE + ACR group compared to rats in the GTE + ACL group, when compared with the positive control group.

The revealed adaptive changes in the biochemical parameters of rat erythrocytes suggest a trigger and (or) enhancing function of exogenous ammonium ions, which mimic the terminal stage of skeletal muscle metabolic acidosis and activates the glycolytic pathway of glucose oxidation in erythrocytes without the participation of the Rapoport–Lubering shunt. Under preconditioning with ammonium salts, the “run-up” of erythrocytes for work under conditions of maximum load occurs not in the skeletal muscles during the performance of this load, but mainly in the peritoneal vessels and portal vein. The Bohr effect is implemented due to the increased lactate (along with carbon dioxide) capacity of red blood cells. The direct effect of ammonium ions on the activity of erythrocyte carbonic anhydrase and the affinity of hemoglobin for oxygen, which does not depend on the effect of pHi, requires special studies.

## 4. Materials and Methods

### 4.1. Chemicals and Green Tea Extract

Ethylenediaminetetraacetic acid (EDTA), ouabain, adenosine triphosphate (ATP), 1,1,3,3-tetraethoxypropane, L-glutathione reduced, glyceraldehyde 3-phosphate (G3P), nicotinamide adenine dinucleotide reduced (NAD^+^) and nicotinamide adenine dinucleotide phosphate (NADP^+^) were purchased from Sigma-Aldrich (St. Louis, MO, USA), while NaCl, KCl, MgCl_2_, CaCl_2_, NaH_2_PO_4_, KH_2_PO_4_, HCl, SnCl_2_, H_2_SO_4_, NaN_3_, hydrogen peroxide, glycine, methanol, ethanol, tris(hydroxymethyl)aminomethane hydrochloride (Tris-HCl), tris(hydroxymethyl)aminomethane (TRIS), trichloroacetic acid (TCA), tris-acetate-EDTA buffer (TAE), sucrose, ammonium orthomolybdate and 5,5′-disulfanediylbis(2-nitrobenzoic acid) (DTNB) were purchased from AO Vekton (St. Petersburg, Russia). The kit for the determination of hemoglobin in blood using the hemoglobin cyanide method was purchased from Syntacon (St. Petersburg, Russia). Lactate dehydrogenase (LDH) and glucose-6-phosphate dehydrogenase (G6PDH) kits were purchased from RANDOX (Crumlin, County Antrim, United Kingdom). Tween 20 was purchased from Ferak Berlin (Berlin, Germany). 1-Chloro-2,4-dinitrobenzene (DNCB) was purchased from Acros Organics BVBA (Geel, Belgium). L-Glutathione oxidized form (glutathione disulfide, GSSG) was purchased from Serva (Heidelberg, Germany).

The green tea extract used was Sunphenon-90D^®^ (Taiyo International, Minneapolis, MN, USA, see Resources—Sunphenon), which, according to the manufacturer’s description, contains catechins with a minimal amount of caffeine: Sunphenon 90D^®^—decaffeinated catechins for use in supplements, foods and beverages with or without tea taste or color; polyphenols > 90%, catechins > 80%, EGCG > 45%, caffeine < 1%. The results of our chemical analysis of ECG are presented in the Supplementary Materials.

### 4.2. Forced Swimming Model and Experimental Scheme

All experiments were carried out in accordance with the rules for conducting work with experimental animals, approved by the ethics commission of the Sechenov Institute of Evolutionary Physiology and Biochemistry of the Russian Academy of Sciences.

**Figure 9:**
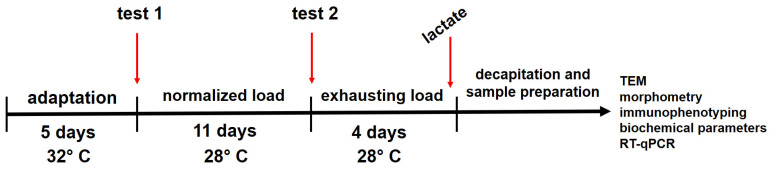
A schematic representation of the experimental design.

Male rats of the outbred Wistar line were kept under standard vivarium conditions. Rats of 8–10 weeks and weighing 210 ± 10 g were previously adapted to water for 5 days at a water temperature of 32 °C, and on the 6th day they were tested with a load of 7% of body weight: swimming for 3 min with an interval of 1 min at a water temperature of 28 °C until complete exhaustion (Figure 9). According to the test results, the rats were divided into groups depending on the duration of swimming, so that the groups did not differ significantly in the average, minimum and maximum duration of swimming. According to the test results, 7 groups were formed: 1st—intact animals (negative control, n = 17); 2nd—positive control: swimming and oral administration of NaCl (saline) at a dose of 10 mg/kg (n = 12) 5 min before exercise; group 3—administration of ACL (ACS reagent, Sigma-Aldrich, USA) at a dose of 20 mg/kg (n = 19) 5 min before the start of exercise; group 4—administration of ACR (ACS reagent, Sigma-Aldrich, USA), at a dose of 10 mg/kg (n = 20) 5 min before the start of exercise; 5th group (GTE, n = 9)—oral administration of an aqueous solution of GTE (decaffeinated green tea extract Sunphenon 90D, Taiyo International Inc., USA; see Supplementary Materials for information on chemical analysis) at a dose of 12 mg/kg in terms of catechins 2 h before exercise, and at 2 h after the end of the load (daily dose of catechins 24 mg/kg); group 6 (GTE + ACL, n = 8)—swimming against the background of GTE (dose and mode of administration—as in group 5) in combination with ACL (10 mg/kg 5 min before the start of the load); group 7 (GTE + ACR, n = 8)—swimming against the background of GTE (dose and mode of administration—as in group 5) in combination with ACR (10 mg/kg 5 min before the start of the load). All drugs were administered starting from the 2nd week of the experiment. During five days of the 2nd week and six days of the 3rd week of the experiment, the rats were subjected to a normalized load: the total duration of swimming was 50–60% of the test indicators. On the sixth day of the 2nd week of the experiment, intermediate testing was performed. During four days of the 4th week of the experiment, the rats were subjected to the maximum load daily.

On the 4th day, 5 min after the end of the load, blood was taken from the tail vein to assess the lactate level, which was determined photometrically using a portable biochemical analyzer Accutrend Plus (Roche Diagnostics GmbH, Mannheim, Germany). One day after the last load, the animals were sacrificed by decapitation, blood was collected in heparinized tubes (final heparin concentration 50 units/mL), and aliquots were selected for hematological and immunological analysis. Hematological analysis was performed on a Medonic M20 analyzer (Boule Medical AB, Spanga, Sweden). After centrifuging the blood at 1500× *g* for 4 min, plasma was collected for biochemical analysis, and the red blood cells were washed twice with cooled saline. Aliquots of plasma and red blood cells were stored at −80 °C until analysis. The SOL and EDL muscles were removed for ultrastructural and morphometric research, RNA isolation and purification.

### 4.3. Peripheral Blood Lymphocyte Immunophenotyping

In brief, 50 μL of rat whole peripheral blood was stained for surface expression with the following specific fluorochrome-conjugated monoclonal antibodies: anti-rat CD44-FITC (cat. No. 103022, clone IM7, Biolegend Inc., San Diego, CA, USA), anti-rat CD49b-PE (cat. No. 108908, clone DX5, Biolegend Inc., USA), anti-rat CD8a-PerCP-Cy5.5 (cat. No. 100734, clone 53-6.7, Biolegend Inc., USA), anti-rat CD4-PE-Cy7 (cat. No. 116016, clone RM4-4, Biolegend Inc., USA), anti-rat CD3-APC (cat. No. 100236, clone 17A2, Biolegend Inc., USA), anti-rat CD45-APC-Cy7 (cat. No. 103116, clone 30-F11, Biolegend Inc., USA), anti-rat CD19-Brilliant Violet 421™ (cat. No. 115538, clone 6D5, Biolegend Inc., USA) and anti-rat CD62L-510™ (cat. No. 104441, clone MEL-14, Biolegend Inc., USA). After incubation at room temperature in the dark for 15 min, erythrocytes were lysed for 15 min with 0.25 mL of OptiLyse C Solution (Beckman Coulter, Inc., Brea, CA, USA). Next, 0.25 mL of sterile PBS was added to each samples; after incubation at room temperature in the dark for 15 min, lymphocyte cell suspensions were washed (7 min 330 g) twice with a buffer (sterile phosphate-buffered saline (PBS) containing 2% heat inactivated fetal bovine serum, Sigma-Aldrich, Burlington, MA, USA) and were resuspended in 0.5 mL of the PBS containing 2% neutral buffered formalin solution (Sigma-Aldrich, USA). Sample acquisition was performed using a CytoFlex cytometer (Beckman Coulter, Inc., USA), equipped with 405, 488 and 638 nm lasers. At least 20000 lymphocytes were analyzed in each sample (doublets were excluded from the analysis using FS-area versus FS-weight dot plot). Obtained data were analyzed with Kaluza software, version 2.0 (Beckman Coulter, Inc., Brea, CA, USA). The data obtained were expressed as a percentage of the population of cells of interest.

### 4.4. Determination of Biochemical Parameters of Blood Plasma

Determination of the main biochemical parameters of blood plasma was carried out on a Sapphire 400 biochemical analyzer (Hirose Electronic System Co., Ltd., Tokyo, Japan) using Randox kits (Randox Laboratories Ltd., Crumlin, UK).

### 4.5. Determination of Biochemical Parameters of Erythrocytes

The concentration of methemoglobin (metHb) was determined according to [117]. ATPase activity was determined according to [118]. EDTA, ATP and ouabain from Sigma were used, and other reagents were from Vecton (St. Petersburg, Russia). The concentration of malondialdehyde (MDA) in erythrocytes was determined by reaction with thiobarbituric acid according to [119], with modifications. 1,1,3,3-tetraethoxypropane (Sigma-Aldrich, Burlington, MA, USA), activity of glutathione peroxidase type 1 (GP1) in erythrocytes (final hemoglobin concentration 300 μg/mL) and glutathione peroxidase type 3 (GPx3) in plasma were used as a standard for constructing a calibration curve. blood (40-fold dilution before reaction) was determined according to Razygraev et al. [120]. Hydrogen peroxide (Vecton, St. Petersburg, Russia) and reduced glutathione (GSH) (Sigma-Aldrich, Burlington, VT, USA) were used. The concentration of total and oxidized glutathione (GSSG) was determined by the Woodward method using metallic zinc as a reducing agent for the GSSG [121], GSH was determined by Ellman’s method [122]. Determination of glyceraldehyde-3-phosphate dehydrogenase (GAPD) activity was carried out spectrophotometrically at +37 °C in a pH 8.9 medium of the following composition, mM: 100 glycine, 100 NaH_2_PO_4_, 5 EDTA, 1 NAD^+^, 1 glyceraldehyde-3-phosphate. The reaction was started by adding hemolysate; the final concentration of hemoglobin (Hb) was 350 μg/mL of the reaction mixture. An increase in the absorption of the mixture was recorded after 2 min at an absorption wavelength of 340 nm on a UV-2401 PC spectrophotometer (Shimadzu, Kyoto, Japan). The total concentration of Hb in washed erythrocytes was determined by the hemoglobin cyanide method (kits from Sintakon, St. Petersburg, Russia). Catalase activity was assessed using the method of Leff et al. [123], glutathione reductase—according to Beutler [124], glutathione-S-transferase—according to Pour et al. [125], activity of LDH—according to Bergmeyer et al. [126], and glucose-6-phosphate dehydrogenase [127] with kits of Randox Laboratories Ltd. (Crumlin, United Kingdom). Transmembrane electron transport (TMET) was measured according to Rizvi et al. [128].

### 4.6. Isolation and Purification of RNA

Cytoplasmic RNA was isolated using the Invitrogen PureLink RNA Mini Kit according to the instructions. To 10 mm^3^ of the sample extracted from the RNALater stabilization solution, 600 μL of 1% 2-mercaptoethanol solution in lysis buffer was added. Muscle homogenization was performed mechanically in the cold using homogenization pestles, followed by centrifugation (2 min, 12,000× *g*, Eppendorf Mini Spin centrifuge, Hauppauge, NY, USA). The supernatant was transferred into RNase-free microtubes, after which extraction was performed with an equal volume of 70% ethanol. The mixture was vortexed to remove visible precipitate, transferred 700 µL at a time to membrane binding columns, and centrifuged (15 s, 12,000× *g*) to remove the aqueous phase. The procedure was repeated if necessary. Purification was carried out using two types of wash buffers, out of the kits for purification. An aliquot of 700 μL of buffer was transferred to the column, after which the aqueous phase was remove by centrifugation (15 s, 12,000× *g*). With the second wash buffer, the washing procedure was repeated twice. Columns with RNA pellet were centrifuged (1 min, 12,000× *g*) to dry the membrane, after which the pellet was dissolved in 40 µL of nuclease-free water (Thermo Scientific, Waltham, MA USA), incubated at room temperature for 1 min, followed by centrifugation (2 min, 12,000× *g*) to elute and collecting the aqueous phase. The concentration of isolated RNA preparations in solution was determined spectrophotometrically (BioSpec Nano, Shimadzu, Kyoto, Japan). The RNA solution was diluted 20-fold in sterile deionized water, after which the spectrum was taken. The purity of the drug was evaluated by the shape of the spectrum and the ratio of absorption at wavelengths of 260 and 280 nm. The frozen RNA solution was stored at −80 °C.

### 4.7. Real-Time PCR after Reverse Transcription

Reverse transcription of 2 µg of the mRNA preparation was performed using the OT-1 reagent kit (Sintol, Moscow, Russia) with oligo(dT)15 primers in accordance with the manufacturer’s recommendations. To normalize the results of real-time PCR after reverse transcription (RT-qPCR), a reference gene was selected from among the constitutively expressed genes used earlier in similar studies: genes for cyclophilin A (*CypA*), beta-actin (*ActB*), phosphoglycerate kinase 1 (*Pgk-1*), hypoxanthine-guanine phosphoribosyltransferase (*HPRT*), ribosomal protein 13A (*Rpl13A*), tyrosine-3-monooxygenase/tryptophan-5-monooxygenase activator protein (*YWHAZ*), TATA-binding protein (*Tbp*).

Primer selection was carried out using the primer-BLAST program, analyzing the NCBI nucleotide sequence databases taking into account the exon-intron structure to exclude genomic DNA amplification (Table 13). For the analysis of candidate reference genes, mRNA preparations from three rats of each experimental group were used. Expression stability was assessed by processing the results of RT-qPCR using geNorm algorithms [129], Normfinder [130], BestKeeper [131] and the comparative method ∆∆Ct [132]. The final processing and ranking of the results was performed using the Reffinder software http://www.heartcure.com.au/reffinder/ or https://blooge.cn/RefFinder/ (accessed on 23 September 2024) [133]. The best expression stability under the experimental conditions of this study were shown by the *Rpl13* and *Tbp* genes, which were used further as reference genes. The target genes of the study were *CASQ1* (skeletal muscle calsequestrin), *SERCA1* (Ca-ATPase of the sarcoplasmic reticulum of fast-twitch muscle fibers), *SERCA2* (Ca-ATPase of the sarcoplasmic reticulum of slow-twitch muscle fibers), *RYR1* (skeletal muscle ryanodine receptor), *CACNA1S* (alpha-1 subunit of dihydropyridine receptor), *PPARGC1A* (PGC-1α, peroxisome proliferator-activated receptor gamma coactivator-1α), as well as myosin heavy chain genes *MYH7* (MyHC1 protein), *MYH2* (MyHC2A), *MYH4* (MyHC2B), *MYH1* (MyHC2X/D). The efficiency of qPCR was evaluated using the method of serial dilutions of cDNA preparations. RT-qPCR results were analyzed using the REST-2009 package [129,134].

### 4.8. Transmission Electron Microscopy (TEM) and Morphometry

To perform TEM, samples of rat muscle tissue were fixed in a 2.5% solution of glutaraldehyde in a cacodylate buffer pH 7.4 containing 0.15 M sucrose, postfixed in a 1% solution of OsO_4_ and, after gradual dehydration in a series of alcohols and acetone, embedded in a mixture of Epon and Araldita. Ultrathin sections were obtained on LKB III (Sweden) and Leica EM UC7 (Austria) ultratomes using glass knives. Sections were placed on copper grids, contrasted with uranyl acetate and lead citrate, and viewed in electron microscopes LIBRA 120 Carl Zeiss (Germany) and FEI Tecnai G2 Spirit BioTWIN (USA). During the morphometric study, only longitudinal sections of myocytes were taken into analysis. For each animal, 20 visual fields were analyzed. All morphometric indicators (average mitochondrial area, mitochondrial volume density, lumen area and number of T-tubules) were determined using the image analysis program “ImageJ” https://imagej.net, using electron diffraction patterns taken at a magnification of 5000×–9000×.

### 4.9. Statistics

Statistical data processing was carried out using Microsoft Excel 2007, STATISTICA 12 and PAST programs https://past.en.lo4d.com/windows. The median, minimum and maximum were calculated for each parameter. Significance of differences was assessed using the Kruskal-Wallis test and post-hoc Dunn test for multiple comparisons. Differences were considered significant at a 95% confidence level (*p* < 0.05).

## 5. Conclusions

In rats of the positive control group (NaCl) adaptation to the increased load actually does not occur. Polyphenols of GTE contributes to the restoration of the body and moderate mobilization of the immune system. Small doses of ammonium salts, especially ammonium carbonate, determine optimal adaptive changes in metabolism and contribute to increased physical performance, which is associated with timely restoration of skeletal muscles and the body as a whole. The combined use of GTE with ammonium salts does not give a clearly positive effect, although there is reason to believe that further search for optimal doses and regimens for using the drugs is promising. The molecular mechanisms of the performance-stimulating effect of small doses of ammonium salts deserve close attention and require further research, as well as schemes for their combined use with green tea polyphenols and other nutraceuticals.

## 6. Limitations of the Research

Large-scale and multifaceted work always raises more questions than it answers. The obvious shortcomings include the use of outbred animals and the uneven number of animals in groups. The large spread of data on many parameters significantly complicated the comparative analysis. An equally significant shortcoming is the forced need to reduce the sample size for morphological and genetic analysis, due to the high cost and labor intensity of these methods for studying biological material. As a result, it is impossible to conduct a full-fledged correlation and multifactorial analysis that would take into account changes in all parameters in all animals in all groups.

Finally, the Sunphenon extract line contains more than 15 products, varying in technology and composition, which are disclosed by the manufacturer only in general terms. We performed a semi-quantitative analysis of the purchased sample for the main polyphenols and caffeine, while such an important component of green tea as theanine, an analogue of the proteinogenic amino acids L-glutamate and L-glutamine, was left out of the analysis. In this regard, for scientific research it is important to either purchase a standardized sample with the results of an accurate quantitative analysis of all the main components according to precise guidelines, or to conduct such an analysis by a scientific institution where the study of the biological activity of a multicomponent extract is carried out.

## Figures and Tables

**Table 1:** Swimming performance of the experimental groups of rats.

Group	Duration of Swimming on the Last Day of the Experiment, Minutes (min; max)	Average Swimming Time, Minutes (min; max)
NaCl (n = 12)	15 (6; 30)	16 (8; 29)
GTE (n = 9)	15 (8; 33)	17 (9; 31)
ACL (n = 19)	24 (6; 51) **	21 (7; 37) *
ACR (n = 20)	27 (9; 67) ***	31 (10; 45) **
GTE + ACL (n = 8)	21 (9; 51) *	19 (9; 33)
GTE + ACR (n = 8)	29 (12; 61) ***	26 (10; 48) **

Notes: *, **, ***—differences from the positive control group (NaCl) are statistically significant (*p* < 0.05, *p* < 0.01, and *p* < 0.001, respectively). Abbreviations: ACL—ammonium chloride; ACR—ammonium carbonate; GTE—green tea extract.

**Table 2:** The concentration of lactate in the blood after exercise in the experimental groups of rats.

Group	Lactate Concentration 5 min after Exercise, mmol/L (min; max)	Lactate Concentration 1 h after Exercise, mmol/L (min; max)
NaCl (n = 12)	7.3 (4.0; 12.0) ***	3.0 (2.1; 4.3)
GTE (n = 9)	6.3 (4.3; 9.5) ***	3.3 (2.3; 3.9)
ACL (n = 19)	6.2 (3.6; 16.9) ***	3.0 (1.9; 4.3)
ACR (n = 20)	5.7 (3.1; 11.7) ***	3.2 (0.8; 5.2)
GTE + ACL (n = 8)	6.1 (3.3; 11.2) ***	3.1 (1.0; 4.0)
GTE + ACR (n = 8)	5.8 (4.1; 8.0) ***	2.8 (0.8; 6.4)

Notes: ***—differences from the negative control group are statistically significant (*p* < 0.001). Abbreviations: ACL—ammonium chloride; ACR—ammonium carbonate; GTE—green tea extract.

**Table 3:** Biochemical parameters of blood plasma on the next day after exercise.

	Control (n = 17)	NaCl (n = 12)	GTE (n = 9)	ACL (n = 19)	ACR (n = 20)	GTE + ACL (n = 8)	GTE + ACR (n = 8)
TP, g/L	65.3 (58.1; 78.6)	61.7 (55.1; 67.3)	66.2 (53.4; 70.4)	65.3 (56.8; 69.3)	63.9 (54.0; 67.0)	64.4 (62.8; 75.9)	64.4 (52.3; 67.3)
ALB, g/L	30.8 (27.6; 33.1)	31.3 (29.8; 32.7)	32.2 (29.4; 33.4)	30.8 (26.8; 33.4)	30.4 (29.0; 32.6)	31.1 (28.9; 33.3)	31.2 (26.6; 32.8)
Glucose, mmol/L	8.5 (7.0; 10.2)	8.6 (6.7; 9.5)	9.1 (8.0; 10.3)	9.0 (7.7; 9.6)	8.1 (6.8; 9.1)	8.1 (6.6; 9.1)	8.2 (6.3; 9.0)
Lactate, mmol/L	1.0 (0.6; 2.3)	0.9 (0.7; 2.3)	0.9 (0.6; 2.5)	1.1 (0.4; 2.2)	0.8 (0.5; 1.1) *	1.0 (0.6; 1.5)	1.0 (0.6; 2.5)
Urea, mmol/L	3.2 (1.6; 5.4)	3.3 (1.3; 4.8)	3.3 (2.0; 6.5)	3.5 (1.8; 5.1)	2.5 (1.7; 3.8) *	3.2 (2.1; 4.1)	2.7 (2.4; 3.6)
Creatinine, µmol/L	43.0 (39.3; 49.0)	38.0 (31.1; 47.0) *	43.0 (29.3; 51.0)	40.3 (35.7; 46.0)	40.5 (35.0; 43.9)	40.0 (24.0; 43.0) *	38.5 (32.0; 48.0) *
ALT, U/L	42.5 (12.6; 104.2)	37.7 (12.6; 71.5)	50.3 (18.9; 97.6)	39.7 (12.6; 125.9)	46.5 (37.4; 107.0)	44.0 (36.9; 52.3)	42.3 (30.2; 53.7)
ALP, U/L	280 (265; 342)	268 (201; 287)	293 (212; 333)	297 (211; 373)	296 (227; 352)	278 (236; 388)	250 (218; 396)
Trigs, mmol/L	1.2 (0.8; 3.6)	1.3 (0.3; 4.5)	1.1 (0.8; 2.6)	1.3 (0.6; 2.3)	1.2 (0.4; 1.5)	1.4 (0.8; 3.7)	0.8 (0.5; 1.2) *
HDL, mmol/L	0.69 (0.30; 0.94)	0.62 (0.25; 0.78)	0.60 (0.40; 0.99)	0.62 (0.27; 0.89)	0.61 (0.40; 0.76)	0.56 (0.28; 0.76)	0.45 (0.26; 0.76) *
LDL, mmol/L	0.46 (0.30; 0.91)	0.53 (0.16; 0.99)	0.46 (0.34; 1.11)	0.40 (0.18; 0.55)	0.40 (0.24; 0.71)	0.43 (0.14; 0.53)	0.37 (0.19; 0.52) ^△^
Chol, mmol/L	1.5 (0.9; 1.8)	1.3 (0.7; 1.6)	1.3 (1.0; 1.8)	1.3 (0.9; 1.9)	1.3 (0.9; 1.7)	1.4 (0.8; 1.7)	1.2 (0.8; 1.6) *
NEFAs, mmol/L	0.70 (0.27; 0.92)	0.58 (0.13; 0.87)	0.53 (0.15; 0.88)	0.54 (0.11; 0.98)	0.53 (0.16; 1.02)	0.64 (0.47; 0.83)	0.59 (0.30; 0.85)
UA, µmol/L	40.0 (19.2; 92.3)	35.4 (20.0; 89.2)	35.4 (20.0; 58.5)	33.0 (24.0; 54.0)	31.7 (19.2; 43.0) *	40.5 (29.0; 81.0)	33.5 (21.0; 43.0)
Phos, mmol/L	2.9 (2.3; 3.2)	2.5 (2.1; 3.5)	2.6 (1.9; 4.7)	2.8 (2.1; 3.4)	2.8 (2.3; 3.2)	3.0 (2.7; 3.2) ^△^	2.8 (2.3; 3.6)
Transf, g/L	1.35 (1.16; 2.08)	1.43 (1.12; 1.83)	1.25 (1.16; 1.89)	1.26 (1.08; 1.85)	1.21 (1.03; 1.15)	1.22 (1.06; 1.50)	1.18 (1.01; 1.38) *
Fe, µmol/L	123.8 (55.8; 171.7)	129.8 (45.7; 301.6)	116.8 (55.8; 154.0)	130.5 (63.4; 195.1)	112.3 (75.4; 160.9)	127.2 (67.9; 172.1)	104.6 (64.5; 173.7)
GPx3, µmol/min×mL	74.6 (44.2; 97.9)	73.6 (51.4; 92.3)	76.1 (57.4; 86.3)	61.0 (47.8; 105.4)	77.6 (55.0; 94.2)	75.9 (55.0; 91.4)	66.9 (55.0; 107.3)
NO_3_ µmol/L	54.2 (44.4; 71.7)	56.2 (38.4; 62.0)	57.6 (39.8; 66.1)	54.5 (38.5; 59.1)	53.0 (38.7; 70.2)	50.7 (45.1; 75.1)	55.8 (50.8; 66.8)
NO_2_ µmol/L	6.3 (0.5; 10.9)	6.2 (2.7; 10.3)	7.9 (4.8; 11.3)	5.7 (1.0; 8.3)	10.3 (1.0; 15.4)	7.5 (4.3; 16.8)	4.3 (2.5; 9.2)

Notes: *—differences from the negative control group are statistically significant (*p* < 0.05); ^△^—differences from the positive control group (NaCl) are statistically significant (*p* < 0.05). Abbreviations: ACL—ammonium chloride; ACR—ammonium carbonate; GTE—green tea extract; TP—total protein; ALB—albumin; ALT—alanine transaminase; ALP—alkaline phosphatase; Trigs—triglycerides; HDL—high-density lipoprotein; LDL—low-density lipoprotein; Chol—cholesterol; NEFAs—non-esterified fatty acids; UA—uric acid; Transf—transferrin; GPx3—glutathione peroxidase 3.

**Table 4:** Biochemical parameters of rat erythrocytes on the next day after exercise.

	Control (n = 17)	NaCl (n = 12)	GTE (n = 9)	ACL (n = 19)	ACR (n = 20)	GTE + ACL (n = 8)	GTE + ACR (n = 8)
GAPD, μmol NAD^+^/min/g_Hb	108.5 (98.8; 151.0)	109.2 (95.7; 138.3)	107.6 (100.5; 114.7)	102.3 (89.2; 129.4)	106.2 (91.7; 123.0)	98.1 (60.2; 104.7) ***	101.2 (89.8; 123.0)
Total glutathione, μmol/g_Hb	9.6 (6.7; 13.1)	9.4 (7.2; 12.8)	9.5 (8.3; 12.8)	9.3 (7.9; 17.4)	8.1 (6.3; 12.0)	8.9 (7.1; 10.5)	9.4 (7.1; 12.0)
GSSG, μmol GSSG/g_Hb	2.7 (0.3; 4.3)	3.1 (1.8; 3.5)	2.9 (1.8; 5.6)	2.6 (0.9; 5.0)	1.8 (0.6; 4.3) ^△^	1.7 (0.2; 5.7)	2.2 (0.8; 3.6)
GSH, μmol/g_Hb	6.2 (4.9; 6.6)	5.8 (2.6; 7.4)	5.5 (4.0; 6.0)	6.3 (4.9; 10.8)	5.5 (1.8; 6.4)	6.0 (3.9; 6.7)	5.7 (4.9; 7.9)
GSH/GSSG	4.4 (2.9; 34.5)	3.5 (1.4; 7.5)	4.2 (1.3; 6.6)	5.0 (3.9; 9.7) ^△^	4.4 (1.0; 13.5)	6.3 (2.2; 49.8) ^△^	4.5 (3.2; 10.3)
MDA, nmol/g_Hb	230.6 (188.8; 411.1)	253.5 (202.8; 293.4)	217.8 (136.4; 324.6)	169.5 (147.9; 202.1) *^△^	183.5 (120.8; 333.3)	224.9 (148.2; 390.1)	193.5 (125.4; 316.2)
Catalase, μmol H_2_O_2_/min/g_Hb	139.0 (113.8; 158.6)	140.3 (120.8; 160.8)	128.4 (115.1; 157.0)	140.0 (121.6; 157.4)	135.9 (111.8; 161.5)	138.3 (122.3; 160.0)	140.4 (123.5; 164.5)
GR, nmol GSH/min/g_Hb	1591.4 (1135.8; 2241.5)	1253.7 (806.4; 1748.3)	941.7 (749.5; 1916.6)	1254.6 (627.8; 3227.3)	1100.8 (459.5; 2398.4) **	1139.5 (402.7; 1992.1) *	1027.1 (701.3; 1608.2) *
GP, μmol GSH/min/g_Hb	678.1 (376.5; 1017.8)	792.5 (514.8; 1093.1)	717.7 (475.2; 1053.3)	883.3 (554.4; 1040.1)	797.5 (485.1; 998.6)	758.8 (639.5; 982.7)	831.0 (464.8; 899.8)
MetHb, %/ g_Hb	0.54 (0.26; 0.67)	0.28 (0.22; 0.62)	0.58 (0.31; 0.76)	0.61 (0.22; 0.70)	0.58 (0.37; 0.67)	0.61 (0.33; 0.74)	0.45 (0.27; 0.76)
GST, μmol GSHconj/min/g_Hb	2.7 (2.4; 3.5)	3.0 (2.6; 3.3)	2.8 (1.0; 3.4)	2.9 (0.9; 3.5)	2.7 (2.1; 3.1)	2.7 (2.5; 3.1)	3.1 (2.3; 3.3)
LDH, μmol/min/g_Hb	85.9 (23.2; 149.0)	81.0 (70.4; 111.4)	104.2 (70.4; 140.4)	78.1 (37.6; 125.9)	83.9 (34.7; 128.8)	100.8 (31.8; 152.9)	80.1 (43.4; 150.5)
G6PD, μmol/min/g_Hb	19.7 (18.8; 24.4)	19.2 (16.9; 23.7)	17.3 (15.9; 22.0)	20.9 (17.8; 27.2)	19.2 (16.4; 40.1)	21.6 (17.6; 28.1)	21.9 (16.9; 25.3)
ATP, μmol/g_Hb	5.8 (4.9; 7.6)	6.4 (5.1; 8.7)	6.1 (2.4; 12.2)	5.5 (1.3; 8.0)	6.1 (2.1; 8.5)	5.4 (3.9; 9.9)	5.4 (4.6; 9.2)
BPG, μmol/g_Hb	39.3 (28.3; 41.5)	37.2 (30.5; 69.0)	34.8 (33.6; 57.0)	40.9 (35.1; 54.4)	43.4 (27.3; 66.3)	50.3 (36.2; 69.6) **	45.2 (27.6; 52.1)
Lactate, μmol/g_Hb	48.0 (31.4; 52.2)	42.4 (37.1; 45.0)	42.7 (25.2; 49.4)	46.5 (29.9; 60.3)	37.7 (28.3; 43.5) **	37.1 (33.7; 52.1) *	41.3 (35.4; 52.5)
Piruvate, μmol/g_Hb	1.4 (0.9; 1.6)	1.2 (0.7; 1.4) *	1.4 (1.1; 2.0)	1.4 (0.7; 1.8)	1.2 (0.9; 1.7)	1.4 (1.1; 1.7) ^△^	1.2 (0.8; 1.5)
Mg^2+^-ATPase, μmol/hr/g_Hb	52.9 (39.6; 62.2)	55.2 (42.8; 66.4)	50.6 (32.4; 60.0)	52.9 (41.0; 68.6)	52.2 (33.9; 70.7)	49.5 (36.5; 53.7)	49.6 (40.8; 56.0)
Na+K+-ATPase, μmol/hr/g_Hb	38.3 (20.6; 64.2)	37.4 (20.4; 55.5)	31.7 (23.0; 58.3)	40.1 (26.4; 49.6)	33.4 (3.0; 57.9)	41.4 (11.9; 66.6)	32.7 (15.3; 45.3)
Ca^2+^-ATPase, μmol/hr/g_Hb	71.7 (34.6; 101.1)	72.0 (49.0; 99.7)	75.4 (26.6; 104.7)	66.1 (26.1; 89.1)	72.8 (47.1; 111.1)	67.9 (49.4; 102.3)	71.7 (31.0; 97.2)
Total ATPase, μmol/hr/g_Hb	145.2 (114.2; 162.9)	147.5 (107.1; 179.1)	142.2 (104.9; 164.0)	131.1 (108.6; 166.8)	137.0 (120.8; 167.1)	139.0 (117.2; 155.2)	129.1 (106.0; 160.5) ^△^
5′-nucleotidase, nmol/min/g_Hb	0.97 (0.37; 1.43)	1.16 (0.53; 1.66)	1.13 (0.60; 1.53)	1.24 (0.82; 2.20)	1.00 (0.70; 1.58)	0.98 (0.44; 1.44)	1.11 (0.59; 1.43)
TMET, nmol/min/g_Hb	167.4 (88.0; 200.0)	107.5 (61.7; 150.0)	75.8 (69.0; 182.1)	68.5 (37.7; 289.6)	86.9 (28.4; 166.4) *	93.9 (59.2; 139.5)	49.5 (34.7; 94.7) *
NO_2_, µmol/L	6.3 (0.5; 10.9)	6.2 (2.7; 10.3)	7.9 (4.8; 11.3)	5.7 (1.0; 8.3)	10.3 (1.0; 15.4)	7.5 (4.3; 16.8)	4.3 (2.5; 9.2)

Notes: *, **, ***—differences from the negative control group are statistically significant (*p* < 0.05, *p* < 0.01, and *p* < 0.001, respectively); ^△^—differences from the positive control group (NaCl) are statistically significant (*p* < 0.05). Abbreviations: ACL—ammonium chloride; ACR—ammonium carbonate; GTE—green tea extract; GAPD—glyceraldehyde-3-phosphate dehydrogenase; GSSG—oxidized glutathione; GSH—reduced glutathione; MDA—malondialdehyde; GR—glutathione reductase; GP—glutathione peroxidase; MetHb—methemoglobin; GST—glutathione-S-transferase; LDH—lactate dehydrogenase; G6PD—glucose-6-phosphate dehydrogenase; BPG—2,3-bisphosphoglycerate; TMET—transmembrane electron transport.

**Table 5:** Hematological parameters of rats on the next day after final exercise load.

	Control (n = 17)	NaCl (n = 12)	GTE (n = 9)	ACL (n = 19)	ACR (n = 20)	GTE + ACL (n = 8)	GTE + ACR (n = 8)
WBC, ×10^9^ cells/L	7.9 (6.0; 11.6)	8.8 (4.9; 12.5)	10.8 (4.9; 16.3)	10.2 (5.3; 16.5)	7.6 (5.2; 14.5)	7.7 (4.0; 13.3)	9.5 (5.1; 10.9)
Lymph, ×10^9^ cells/L	5.6 (4.6; 7.7)	6.1 (3.5; 10.4)	7.7 (3.6; 11.8) *	7.5 (3.6; 11.3)	5.9 (3.5; 10.0)	5.5 (1.9; 8.9)	6.4 (4.0; 8.6)
Lymph %	69.8 (57.7; 79.7)	72.5 (60.7; 82.7)	72.4 (60.7; 79.7)	73.1 (61.6; 80.9)	74.4 (68.0; 81.1)	69.3 (47.7; 72.2)	77.8 (55.3; 84.3)
Gran, ×10^9^ cells/L	1.8 (0.3; 4.2)	1.8 (1.1; 3.4)	2.5 (0.2; 4.7)	2.4 (1.4; 4.1)	1.6 (0.2; 2.7)	1.9 (0.7; 4.0)	1.9 (0.3; 3.3)
Gran %	23.4 (5.1; 37.9)	21.9 (14.0; 36.0)	23.4 (5.1; 36.1)	23.5 (16.8; 32.6)	19.6 (3.3; 25.4)	25.6 (10.9; 48.5)	17.8 (6.2; 32.7)
RBC, ×10^12^ cells/L	7.3 (6.4; 8.3)	7.5 (5.2; 8.3)	7.7 (5.7; 8.8)	7.3 (6.3; 8.3)	7.6 (6.6; 8.4)	6.8 (6.0; 8.6)	6.8 (6.4; 8.0)
Hb, g/L	131 (120; 172)	125 (100; 137)	128 (105; 151)	130 (114; 152)	132 (124; 157)	128 (119; 153)	126 (119; 138)
HCT %	39.7 (30.8; 49.5)	39.9 (25.5; 45.7)	40.7 (31.5; 45.6)	38.7 (31.9; 46.8)	41.5 (32.6; 45.5)	34.3 (31.8; 45.4)	35.4 (31.5; 39.8)
MCV, fL	53.3 (47.9; 61.8)	53.9 (49.4; 56.0)	52.2 (47.1; 56.0)	53.1 (45.6; 58.5)	53.3 (47.1; 57.4)	52.4 (47.4; 53.4)	50.1 (49.2; 55.4)
MCH, pg	19.0 (18.2; 21.4)	18.7 (18.1; 19.9)	18.4 (17.8; 20.8)	18.4 (17.4; 19.5)	18.5 (17.8; 19.5)	18.5 (17.4; 20.1)	18.8 (17.2; 19.1)
MCHC, g/L	344 (339; 350)	343 (336; 356)	345 (333; 355)	335 (324; 347)	340 (326; 345)	340 (336; 350)	341 (332; 346)
RDW, fL	20.0 (15.7; 22.1)	18.5 (15.2; 19.8)	18.8 (14.9; 22.4)	19.3 (14.4; 21.8)	18.7 (14.2; 21.6)	17.6 (15.1; 20.4)	17.7 (12.6; 20.2) *
PLT, ×10^9^ cells/L	708 (362; 1480)	634 (436; 965)	475 (107; 919)	531 (241; 2536) *	524 (422; 1114)	580 (346; 847)	579 (264; 770) *
MPV, fL	5.9 (5.4; 6.4)	5.9 (5.2; 7.1)	5.7 (5.4; 7.3)	6.2 (5.5; 7.1)	6.1 (5.5; 6.5)	6.0 (5.7; 6.4)	6.0 (5.1; 6.9)
PDW, fL	16.8 (15.5; 17.6)	17.0 (16.3; 17.9)	17.1 (15.9; 17.9)	17.4 (16.6; 19.9) **	17.2 (15.5; 17.9)	16.9 (16.3; 17.7)	17.0 (16.4; 18.8)
PCT %	0.44 (0.20; 0.86)	0.35 (0.30; 0.60)	0.32 (0.07; 0.54)	0.29 (0.06; 0.43) **^△^	0.33 (0.11; 0.60) *	0.36 (0.22; 0.52)	0.33 (0.18; 0.49)

Notes: *, **—differences from the negative control group are statistically significant (*p* < 0.05 and *p* < 0.01, respectively); ^△^—differences from the positive control group (NaCl) are statistically significant (*p* < 0.05). Abbreviations: ACL—ammonium chloride; ACR—ammonium carbonate; GTE—green tea extract; WBC—white blood cell count; Lymph—lymphocytes; Gran—granulocyte; RBC—red blood cell count; Hb—hemoglobin; HCT—hematocrit; MCV—mean corpuscular volume; MCH—mean concentration hemoglobin; MCHC—mean corpuscular hemoglobin concentration; RDW—red blood cell distribution width; PLT—platelet count; MPV—mean platelet volume; PDW—platelet distribution width; PCT—plateletcrit.

**Table 6:** Immunological parameters of rat erythrocytes on the next day after final exercise load.

	Control (n = 17)	NaCl (n = 12)	GTE (n = 9)	ACL (n = 19)	ACR (n = 20)	GTE + ACL (n = 8)	GTE + ACR (n = 8)
B-cells	1.2 (0.9; 2.9)	1.7 (0.7; 2.5)	1.6 (0.9; 3.0)	1.9 (0.9; 3.1) **	1.5 (0.8; 3.7)	1.6 (0.5; 2.2)	1.7 (1.0; 2.7)
Th %	68.7 (47.1; 83.7)	77.3 (56.4; 85.6)	73.4 (47.5; 78.1)	73.6 (68.8; 83.7)	66.6 (39.7; 81.6)	71.4 (57.8; 84.5)	66.3 (45.0; 76.1) ^△△^
Th	2.6 (1.8; 4.4)	3.2 (1.3; 6.3)	4.2 (1.7; 5.7) *	3.7 (1.5; 6.0)	2.5 (1.6; 5.3)	2.6 (1.2; 3.8)	2.9 (1.4; 4.7)
Tcyt %	29.8 (15.0; 51.6)	21.4 (12.6; 42.6)	25.0 (20.6; 51.4)	25.1 (15.0; 30.0)	32.2 (17.0; 59.0)	27.2 (14.1; 40.4)	32.2 (22.8; 52.6) ^△^
Tcyt	1.13 (0.68; 2.57)	0.88 (0.68; 1.50)	1.48 (0.52; 2.70) ^△^	1.19 (0.54; 1.76)	1.19 (0.48; 2.57)	1.01 (0.20; 2.55)	1.37 (1.15; 2.17) ^△^
NKT-cells %	8.2 (1.0; 27.1)	2.4 (1.7; 22.0) *	3.8 (2.6; 25.9)	2.6 (1.6; 7.1) *	5.5 (1.7; 29.6)	4.8 (2.4; 8.9)	10.6 (2.1; 25.8) ^△^
T-cells	3.9 (3.2; 5.2)	4.2 (2.3; 7.7)	5.5 (2.3; 8.3) *	4.8 (2.1; 7.7)	3.6 (2.5; 6.6)	3.7 (1.4; 6.4)	4.2 (2.7; 6.1)
CD4/CD8	2.4 (0.9; 5.3)	3.4 (1.3; 6.2)	2.8 (1.0; 3.7)	2.9 (2.3; 5.4)	2.1 (0.7; 4.7)	2.6 (1.5; 6.0)	2.1 (1.0; 3.3) ^△^
CD4-CD8-	0.024 (0.010; 0.050)	0.024 (0.011; 0.042)	0.029 (0.023; 0.068) *	0.026 (0.014; 0.065)	0.023 (0.014; 0.058)	0.021 (0.008; 0.054)	0.031 (0.025; 0.061)
Tcyt 62L− %	34.5 (3.4; 81.2)	9.4 (3.3; 83.1)	17.3 (6.2; 69.2)	14.5 (4.2; 43.8)	26.9 (4.7; 89.1)	33.6 (4.1; 45.3)	49.7 (10.4; 81.0) ^△^
Tcyt 62L−	0.49 (0.03; 2.09)	0.15 (0.03; 0.82)	0.24(0.07; 1.95)	0.14 (0.03; 0.79)	0.25 (0.03; 2.08)	0.28 (0.05; 1.01)	0.69 (0.12; 1.35) ^△^
Tcyt 62L+ %	65.5 (18.8; 96.7)	90.6 (16.9; 96.7)	82.7 (30.8; 93.9)	85.5 (56.2; 95.9)	73.1 (10.9; 95.3)	66.4 (54.8; 95.9)	50.3 (19.0; 89.7) ^△^
Tcyt 62L+	0.68 (0.41; 0.93)	0.62 (0.17; 1.26)	0.92 (0.32; 1.66) *	1.01 (0.47; 1.28) *	0.81 (0.25; 1.26)	0.68 (0.13; 1.59)	0.76 (0.31; 1.20)
Tcyt 44+62L+ %	7.1 (4.2; 10.9)	10.0 (2.8; 15.0)	7.3 (3.1; 15.1)	8.0 (3.3; 17.7)	9.5 (1.0; 15.4)	7.7 (3.7; 12.7)	5.5 (2.1; 14.9) ^△^
Tcyt 44dim62L− %	30.0 (2.9; 72.6)	8.2 (2.6; 77.2)	12.0 (5.1; 59.2)	10.9 (3.3; 38.4)	22.9 (3.3; 84.1)	28.8 (2.8; 38.6)	46.1 (7.4; 73.1) ^△△^
Tcyt 44dim62L−	0.38 (0.02; 1.87)	0.13 (0.02; 0.76)	0.18 (0.05; 1.67)	0.12 (0.02; 0.69)	0.21 (0.02; 1.96)	0.24 (0.03; 0.90)	0.62 (0.09; 1.26) ^△^
Tcyt 44dim62L+ %	56.4 (14.6; 87.2)	78.6 (14.1; 86.7)	74.1 (26.8; 81.8)	76.6 (50.9; 84.8)	65.6 (9.8; 83.5)	60.7 (46.9; 83.2)	46.3 (15.9; 80.5) ^△^
Tcyt 44dim62L+	0.60 (0.36; 0.80)	0.55 (0.14; 1.13)	0.86 (0.28; 1.44) *	0.87 (0.42; 1.13) *	0.71 (0.23; 1.06)	0.61 (0.12; 1.49)	0.70 (0.25; 1.11)
Th 62L− %	6.4 (3.6; 17.9)	7.5 (4.0; 13.5)	7.6 (5.3; 20.7)	8.9 (3.7; 14.1)	10.8 (4.1; 17.8)	9.4 (6.1; 24.2) *	10.4 (4.3; 15.5) *
Th 62L−	0.16 (0.11; 0.47)	0.23 (0.12; 0.53)	0.32 (0.13; 0.89) *	0.31 (0.13; 0.56) *	0.25 (0.09; 0.48)	0.24 (0.15; 0.66)	0.29 (0.14; 0.47)
Th 62L+ %	93.6 (82.1; 96.4)	92.6 (86.5; 96.1)	92.4 (79.3; 94.7)	91.1 (85.9; 96.3)	89.2 (82.2; 95.9)	90.6 (75.8; 93.9) *	89.6 (84.5; 95.7) *
Th 44+62L− %	3.2 (1.8; 10.4)	4.5 (2.1; 8.7)	4.6 (2.8; 14.6)	5.2 (1.7; 7.9)	6.1 (2.5; 9.3) *	6.2 (3.3; 14.0) **	6.3 (2.2; 10.3) *
Th 44+62L−	0.09 (0.05; 0.25)	0.13 (0.07; 0.29)	0.19 (0.07; 0.63)	0.19 (0.08; 0.39) *	0.18 (0.06; 0.29)	0.16 (0.10; 0.37)	0.16 (0.07; 0.27)
Th 44dim62L+ %	80.3 (67.2; 87.9)	80.7 (73.4; 87.1)	80.5 (69.1; 84.5)	79.3 (73.2; 88.9)	76.8 (70.7; 84.2)	74.2 (64.7; 79.5) **^△△^	75.8 (70.5; 81.3)

Notes: *, **—differences from the negative control group are statistically significant (*p* < 0.05 and *p* < 0.01, respectively); ^△^, ^△△^—differences from the positive control group (NaCl) are statistically significant (*p* < 0.05 and *p* < 0.01, respectively). Abbreviations: ACL—ammonium chloride; ACR—ammonium carbonate; GTE—green tea extract; Th—T helper cells; Tcyt—cytotoxic T cells; NKT—natural killer T cells.

**Table 7:** Expression of SOL genes. Data are presented as expression relative to the negative control group (SE, standard error, in brackets).

	NaCl (n = 8)	GTE (n = 9)	ACL (n = 9)	ACR (n = 9)	GTE + ACL (n = 8)	GTE + ACR (n = 8)
*Cacna*	0.744 (0.348–1.303)	0.869 (0.381–1.849)	1.000 (0.540–2.395)	1.393 (0.749–2.628)	1.087 (0.620–2.203)	1.374 (0.744–2.347)
*RYR1*	0.874 (0.513–1.511)	0.822 (0.420–1.579)	1.308 (0.659–2.926)	1.273 (0.804–2.344)	1.101 (0.669–1.940)	0.959 (0.664–1.455)
*Casq1*	0.773 (0.474–1.532)	0.728 (0.329–1.746)	1.363 (0.709–2.749)	1.724 * (0.979–3.158)	2.091 *** (1.315–3.137)	1.521 (0.917–2.610)
*Serca1*	0.636 (0.243–1.823)	0.501 (0.062–2.404)	1.187 (0.609–2.263)	1.195 (0.557–2.560)	1.121 (0.557–2.366)	1.096 (0.640–2.030)
*Serca2*	1.631 (0.464–2.796)	1.488 (0.428–2.070)	3.013 * (1.044–3.269)	3.573 ** (1.200–3.992)	3.331 * (0.877–78.520)	3.127 * (0.933–46.857)
*PGC*	0.819 (0.445–1.468)	0.741 (0.305–1.500)	1.090 (0.630–1.712)	0.754 (0.459–1.258)	0.827 (0.537–1.487)	0.587 ** (0.406–0.808)
*Myhc1*	0.567 (0.091–2.154)	0.472 (0.101–1.639)	5.644 * (1.387–28.379)	0.929 (0.193–3.914)	0.614 (0.122–2.713)	2.156 (0.291–20.367)
*Myhc2a*	0.153 (0.022–1.872)	0.496 (0.045–4.171)	1.318 (0.320–3.296)	0.947 (0.267–3.543)	0.169 * (0.029–1.193)	0.416 (0.071–2.248)
*Myhc2b*	0.802 (0.132–4.792)	1.666 (0.134–12.406)	0.613 (0.068–3.447)	0.786 (0.149–5.522)	1.687 (0.146–14.481)	1.441 (0.130–19.267)
*Myhc2xd*	0.531 (0.312–2.127)	1.813 (0.362–8.460)	0.378 (0.083–1.426)	0.920 (0.257–2.997)	1.144 (0.350–3.100)	4.252 * (0.995–50.162)

*, **, ***—differences from the negative control group are statistically significant (*p* < 0.05, *p* < 0.01, and *p* < 0.001, respectively).

**Table 8:** Expression of EDL genes. Data are presented as expression relative to the negative control group (SE, standard error, in brackets).

	NaCl (n = 8)	GTE (n = 9)	ACL (n = 9)	ACR (n = 9)	GTE + ACL (n = 8)	GTE + ACR (n = 8)
*Cacna*	0.597 (0.327–1.058)	0.708 (0.357–1.533)	0.960 (0.265–2.926)	1.615 (0.920–3.593)	0.907 (0.482–1.597)	1.245 (0.564–2.493)
*RYR1*	0.824 (0.501–1.311)	0.799 (0.498–1.328)	0.923 (0.317–2.320)	1.241 (0.699–2.291)	0.971 (0.562–1.693)	0.941 (0.579–1.619)
*Casq1*	0.597 (0.357–0.970)	0.910 (0.197–2.279)	1.240 (0.385–2.541)	1.843 * (1.204–3.046)	1.656 (0.665–2.503)	1.125 (0.413–2.441)
*Serca1*	0.830 (0.423–1.624)	0.970 (0.543–1.899)	0.863 (0.333–2.450)	1.375 (0.640–2.484)	0.963 (0.587–1.839)	0.354 (0.139–2.022)
*Serca2*	3.180 * (1.122–10.418)	1.261 (0.461–4.134)	3.400 * (1.013–12.443)	2.044 (0.807–9.180)	2.611 * (0.908–8.921)	4.514 * (1.142–23.062)
*PGC*	1.560 (0.888–2.014)	0.842 (0.525–1.097)	1.093 (0.628–1.710)	1.589 ** (1.088–3.007)	1.005 (0.695–1.523)	1.130 (0.697–1.989)
*Myhc1*	7.017 ** (1.762–24.586)	0.747 (0.166–5.452)	2.537 (0.107–47.411)	1.158 (0.077–21.211)	1.817 (0.462–18.654)	12.324 * (1.309–101.184)
*Myhc2a*	0.924 (0.569–1.390)	0.593 (0.241–1.112)	1.086 (0.437–3.951)	0.950 (0.410–2.477)	1.216 (0.457–2.577)	1.063 (0.514–2.691)
*Myhc2b*	0.619 (0.261–1.459)	1.029 (0.526–3.033)	1.748 (0.762–4.270)	1.767 (0.562–4.506)	1.321 (0.555–3.679)	1.200 (0.543–3.415)
*Myhc2xd*	1.230 (0.805–1.983)	2.213 * (0.869–5.003)	1.264 (0.696–2.504)	1.797 * (0.978–3.367)	1.728 (0.815–5.628)	1.212 (0.404–2.999)

*, **—differences from the negative control group are statistically significant (*p* < 0.05 and *p* < 0.01, respectively).

**Table 9:** Morphometric analysis of slow-twitch SOL muscles.

Group	Average Mitochondrial Area (um^2^ ± SEM)	Average Cross-Sectional Area of T-Tubules (nm^2^ ± SEM)	Average Area of Longitudinal Section of T-Tubules (um^2^ ± SEM)
Control (n = 7)	0.057 (±0.001)	3330 (±200)	9254 (±460)
NaCl (n = 8)	0.065 (±0.001)	2070 (±20) ***	11,000 (±570)
GTE (n = 9)	0.064 (±0.005)	1620 (±990) ***	10,636 (±1600)
ACL (n = 9)	0.068 (±0.001) *	2400 (±40) **	11,895 (±600)
ACR (n = 8)	0.069 (±0.001) ***	2520 (±20) *	12,912 (±510) *
GTE + ACL (n = 8)	0.118 (±0.003) *** ^△△△^	2970 (±40) ^△△^	18,590 (±580) *** ^△△△^
GTE + ACR (n = 8)	0.071 (±0.002) **	2520 (±70) *	10,142 (±820)

Notes: *, **, ***—differences from the negative control group are statistically significant (*p* < 0.05, *p* < 0.01 and *p* < 0.001, respectively); ^△△^, ^△△△^—differences from the positive control group (NaCl) are statistically significant (*p* < 0.01 and *p* < 0.001, respectively).

**Table 10:** Morphometric analysis of fast-twitch EDL muscles.

Group	Average Mitochondrial Area (um^2^ ± SEM)	Average Cross-Sectional Area of T-Tubules (nm^2^ ± SEM)	Average Area of Longitudinal Section of T-Tubules (nm^2^ ± SEM)
Control (n = 7)	0.053 (±0.002)	2370 (±50)	9687 (±680)
NaCl (n = 8)	0.086 (±0.003) ***	1850 (±20)	12,345 (±470)
GTE (n = 9)	0.046 (±0.002) ^△△△^	2060 (±80)	9490 (±680)
ACL (n = 9)	0.099 (±0.003) ***	2180 (±10)	14,284 (±370) ***
ACR (n = 8)	0.099 (±0.002) ***	3610 (±140) *** ^△△△^	17,270 (±580) *** ^△△△^
GTE+ACL (n = 8)	0.055 (±0.003) ^△△△^	2910 (±70) ^△△△^	15,145 (±450) ***^△^
GTE + ACR (n = 8)	0.109 (±0.003) *** ^△△^	2900 (±40) ^△△△^	17,129 (±630) *** ^△△△^

Notes: ***—differences from the negative control group are statistically significant (*p* < 0.001); ^△^, ^△△^, ^△△△^—differences from the positive control group (NaCl) are statistically significant (*p* < 0.05, *p* < 0.01 and *p* < 0.001, respectively).

**Table 11:** Changes in the indicators of rats of different groups relative to the indicators of the negative control group 5 min (lactate) and 24 h after the end of the last load and the forced swimming cycle.

	NaCl (n = 12)	GTE (n = 9)	ACL (n = 19)	ACR (n = 20)	GTE + ACL (n = 8)	GTE + ACR (n = 8)
Lactate concentration 5 min after exercise	↑↑	↑↑	↑↑	↑↑	↑↑	↑↑
Biochemical parameters of blood plasma	Crea ↓			Urea ↓ UA ↓ Lactate ↓	Crea ↓	Transf ↓ Trigs ↓ Crea ↓ HDL ↓ Chol ↓
Biochemistry of red blood cells	Piruvate ↓		MDA ↓	GR ↓ Lactate ↓ TMET ↓↓	GAPD ↓ GR ↓ BPG ↑ Lactate ↓	GR ↓ TMET ↓↓
Hematological parameters		Lymph ↑	PLT ↓ PDW ↓ PCT% ↓	PCT% ↓		RDW ↓ PLT ↓
Immunological parameters	NKT-cells% ↓↓	T-cells ↑ Th ↑↑ Th 62L− ↑↑ CD4-CD8- ↑ Tcyt 62L+ ↑ Tcyt 44dim62L+ ↑	B-cells ↑↑ NKT-cells% ↓↓ Tcyt 62L+ ↑ Tcyt 44dim62L+ ↑ Th 62L− ↑↑ Th 44+62L− ↑↑	Th 44+62L− % ↑↑	Th 62L− % ↑ Th 62L+ % ↓ Th 44+62L− % ↑↑ Th 44dim62L+ % ↓	Th 62L− % ↑↑ Th 62L+ % ↓ Th 44+62L− % ↑↑
Genes of EDL muscles	*Myhc1* ↑↑ *Serca2* ↑↑	*Myh2xd* ↑↑	*Serca2* ↑↑	*Myh2xd* ↑↑ *CASQ1* ↑↑ *PGC1* ↑↑	*Serca2* ↑↑	Myhc1 ↑↑ Serca2 ↑↑
Genes of SOL muscles			*Myhc1* ↑↑ *Serca2* ↑↑	*CASQ1* ↑↑ *Serca2* ↑↑	*Myhc2a* ↓↓ *CASQ1* ↑↑ *Serca2* ↑↑	*Myh2xd* ↑↑ *Serca2* ↑↑ *PGC1* ↓↓
Morphometry of EDL muscles	Average mitochondrial area ↑↑		Average mitochondrial area ↑↑ Average area of longitudinal section of T-tubules ↑	Average mitochondrial area ↑↑ Average cross-sectional area of T-tubules ↑↑ Average area of longitudinal section of T-tubules ↑↑	Average area of longitudinal section of T-tubules ↑↑	Average mitochondrial area ↑↑ Average area of longitudinal section of T-tubules ↑↑
Morphometry of SOL muscles	Average cross-sectional area of T-tubules ↓	Average cross-sectional area of T-tubules ↓↓	Average mitochondrial area ↑ Average cross-sectional area of T-tubules ↓	Average mitochondrial area ↑ Average cross-sectional area of T-tubules ↓ Average area of longitudinal section of T-tubules ↑	Average mitochondrial area ↑↑ Average area of longitudinal section of T-tubules ↑↑	Average mitochondrial area ↑↑ Average cross-sectional area of T-tubules ↓

↑ the value is statistically significantly increased, but relatively weakly (up to 50%); ↑↑ the value is statistically significantly and strongly increased (more than 50%); ↓ the value is statistically significantly reduced, but relatively weakly (up to 50%); ↓↓ the value is statistically significantly and strongly reduced (more than 50%).

**Table 12:** Changes in the indicators of rats of different groups relative to the indicators of the positive control group 24 h after the last load and the forced swimming cycle.

	GTE (n = 9)	ACL (n = 19)	ACR (n = 20)	GTE + ACL (n = 8)	GTE + ACR (n = 8)
Duration of swimming		Last day ↑↑ Average time ↑	Last day ↑↑ Average time ↑↑	Last day ↑	Last day ↑↑ Average time ↑↑
Biochemical parameters of blood plasma				Phos ↑	LDL ↓
Biochemistry of red blood cells		GSH/GSSG ↑ MDA ↓	GSSG ↓	GSH/GSSG ↑↑ Piruvate ↑	Total ATPase ↓
Hematological parameters		PCT% ↓			
Immunological parameters	Tcyt ↑↑			Th 44dim62L+% ↓	Th % ↓ Tcyt % ↑ Tcyt ↑↑ NKT-cells% ↑↑ CD4/CD8 ↓ Tcyt 62L− % ↑↑ Tcyt 62L+ % ↓ Tcyt 62L− ↑↑ Tcyt 44+62L+ % ↓ Tcyt 44dim62L− % ↑↑ Tcyt 44dim62L− ↑↑ Tcyt 44dim62L+ % ↓
Morphometry of EDL muscles	Average mitochondrial area ↓		Average cross-sectional area of T-tubules ↑↑ Average area of longitudinal section of T-tubules ↑	Average mitochondrial area ↓↓ Average cross-sectional area of T-tubules ↑↑ Average area of longitudinal section of T-tubules ↑↑	Average mitochondrial area ↑ Average cross-sectional area of T-tubules ↑↑ Average area of longitudinal section of T-tubules ↑
Morphometry of SOL muscles				Average mitochondrial area ↑↑ Average cross-sectional area of T-tubules ↑ Average area of longitudinal section of T-tubules ↑↑	

↑ the value is statistically significantly increased, but relatively weakly (up to 50%); ↑↑ the value is statistically significantly and strongly increased (more than 50%); ↓ the value is statistically significantly reduced, but relatively weakly (up to 50%); ↓↓ the value is statistically significantly and strongly reduced (more than 50%).

**Table 13:** Target genes, primer and probe sequences.

Gene	Forward Primer	Reverse Primer	Probe
*CASQ1*	5′-ACCTTCCTACCG-CCCATG-3′	5′-ATGTAGACCACAG-TTGGCCCTATAGTA-3′	5′-CCGAGCTCCTTG-GGACACTAGGTCATTC-3′
*CASQ2*	5′-CAGAATATTACA-AAGCGTTCCAAG-AG-3′	5′-CAACTTCTTTGCCA-CCCCC-3′	5′-CCGAGCTCCTTG-GGACACTAGGTCAT-TC-3′
*SERCA1*	5′-GGTTTGGCAGGA-ACGGAAT-3′	5′-GGTGGATTTGATG-GAGAGGAT-3′	5′-CGATGTCCCGAG-CCT-TGATCC-3′
*SERCA2*	5′-AGTGGCTGATG-GTGCTGAAA-3′	5′-GCACCCGAACACC-CTTACAT-3	5′-TTACTCCAGTATT-GCAGGCTCCAGGTA-3′
*RyR1*	5′-TCATCGTCAATA-ACCTGGGCATC-3′	5′-CTTACGCAGTCG-CCCGATA-3′	5′-ACACAGCCAACC-GCTT-CATCCAC-3′
*CACNA1*	5′-CATGCAGATGT-TCGGGAAGA-3′	5′-GCTTGTGGGAAAG-TCTGGAAGT-3′	5′-CCATGGTGGACG-GGACGCAAATAAA-3′
*MYHC1*	5′-AGACAGAGAAT-GGCAAGACG-3′	5′-GGTGTAGATCATCC-AGGAAGCG-3′	5′-TTGTCGAACTTG-GGAGGGTTCTGC-3′
*MYHC2x/d*	5′-CGAGGAAGCG-GAGGAACAAT-3′	5′-TGGATCGATCACTC-TTCGCT-3′	5′-AAGTTCCGCAAG-ATCCAGCACGA-3′
*MYHC2a*	5′-GGAGGCTGAGG-AACAATCCA-3′	5′-CAGAGCTGCCTTA-CTCTTCACT-3′	5′-TCTATCCAAGTT-CCGCAAGCTGCA-3′
*MYHC2b*	5′-ACACACAGAGT-CAGGTGAGTT-3′	5′-TGGCCTTGGACTC-TTCTTCTAG-3′	5′TCTCCCGAGGCA-AACAAGCGTTTA-3′
*PGC1a*	5′-GTGCAGCCAAG-ACTCTGTATGG-3′	5′GTCCAGGTCATTCACATCAAGTTC-3′	5′-AACCAGGGCAGC-ACACTCTATGTC-3′

## Data Availability

The data presented in this study are available from the corresponding authors upon reasonable request.

## Supplementary Materials

The supporting information can be downloaded at: https://www.mdpi.com/article/10.3390/ijms251910438/s1.

## Abbreviations

ACL	ammonium chloride
ACR	ammonium carbonate
ALB	Albumin
ALP	alkaline phosphatase
ALT	alanine transaminase
AMPK	5′-adenosine monophosphate-activated protein kinase
ATP	adenosine triphosphate
AQ	aquaporins
BPG	2,3-bisphosphoglycerate
CACNA1S	dihydropyridine receptor, L-type calcium channel
CASQ	calsequestrin
Chol	Cholesterol
CICR	calcium-induced calcium release
CYB5R	cytochrome-b5 reductase
DICR	depolarization-induced calcium release
DNT cells	double-negative T cells
EC	Epicatechin
ECC	excitation–contraction coupling
ECG	epicatechin gallate
EGC	Epigallocatechin
EGCG	epigallocatechin-3-gallate
EDL	m. extensor digitorum longus
EDTA	ethylenediaminetetraacetic acid
EMC	electromechanical coupling
GAPD	glyceraldehyde-3-phosphate dehydrogenase
GP	glutathione peroxidase
G6PD	glucose-6-phosphate dehydrogenase
GR	glutathione reductase
Gran	Granulocyte
GSH	reduced glutathione
GSSG	oxidized glutathione
GST	glutathione-S-transferase
GTE	green tea extract
Hb	Hemoglobin
HCT	Hematocrit
HDL	high-density lipoprotein
HNE	4-hydroxy-2-nonenal
LDH	lactate dehydrogenase
LDL	low-density lipoprotein
Lymph	Lymphocytes
metHb	Methemoglobin
MCH	mean concentration hemoglobin
MCHC	mean corpuscular hemoglobin concentration
MCT	monocarboxylate transporter
MCV	mean corpuscular volume
MDA	Malondialdehyde
MPV	mean platelet volume
MRP1	multidrug resistance protein 1
MyHC	myosin heavy chain
NEFAs	non-esterified fatty acids
NF-κB	nuclear factor kappa-light-chain-enhancer of activated B cells
NK	natural killer cells
NKT	natural killer T cells
PCT	Plateletcrit
PDW	platelet distribution width
PEPCK	phosphoenolpyruvate carboxykinase
PGC-1α	peroxisome proliferator-activated receptor gamma coactivator-1α
PLT	platelet count
PPAR	peroxisome proliferator-activated receptor
RBC	red blood cell count
RDW	red blood cell distribution width
ROS	reactive oxygen species
RYR	ryanodine receptor
SB	sodium bicarbonate
SC	sodium citrate
SERCA	calcium ATPase
SOL	m. soleus
SR	sarcoplasmic reticulum
TCR	T-cell receptor
Tcyt	cytotoxic T cells
TEM	transmission electron microscopy
Th	T helper cells
TMET	transmembrane electron transport
TP	total protein
Transf	Transferrin
Tregs	regulatory T cells
Trigs	Triglycerides
UA	uric acid
WBC	white blood cell count
