# Supplementary material for: Testing Green Tea Extract and Ammonium Salts as Stimulants of Physical Performance in a Forced Swimming Rat Experimental Model
Source: Int J Mol Sci. 2024 Sep 27;25(19):10438. doi: 10.3390/ijms251910438 (PMC11477139; doi:10.3390/ijms251910438)
Supplement: Supplementary file 1 [file ijms-25-10438-s001.zip › ijms-3153770-supplementary.pdf]

Supplement.

Sunphenon® is a line of innovative extracts from Taiyo (Yokkaichi, Japan), the production methods of which provide high levels of polyphenols (catechins) (Table S1).

Table S1. Components Contained in Green Tea Extracts

Component	Formula	Mass spectroscopic characteristics
Epigallocatechin gallate	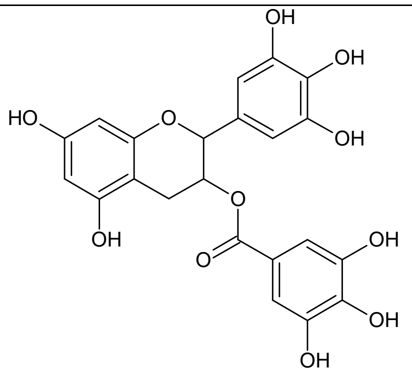	Molecular Formula: $C_{22}H_{18}O_{11}$<br>Formula Weight: 458.37172<br>Monoisotopic Mass: 458.084911 Da<br>[M+H]+: 459.092188 Da<br>[M-H]-: 457.077635 Da
Gallic acid (Gallate)	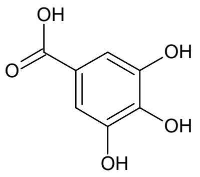	Molecular Formula: $C_7H_6O_5$<br>Formula Weight: 170.11954<br>Monoisotopic Mass: 170.021523 Da<br>[M+H]+: 171.0288 Da<br>[M-H]-: 169.014247 Da
Epigallocatechin	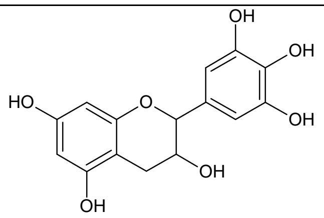	Molecular Formula: $C_{15}H_{14}O_7$<br>Formula Weight: 306.26746<br>Monoisotopic Mass: 306.073953 Da<br>[M+H]+: 307.081229 Da<br>[M-H]-: 305.066676 Da
Epicatechin	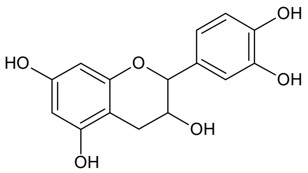	Molecular Formula: $C_{15}H_{14}O_6$<br>Formula Weight: 290.26806<br>Monoisotopic Mass: 290.079038 Da<br>[M+H]+: 291.086315 Da<br>[M-H]-: 289.071762 Da
Epicatechin gallate	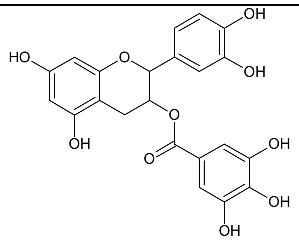	Molecular Formula: $C_{22}H_{18}O_{10}$<br>Formula Weight: 442.37232<br>Monoisotopic Mass: 442.089997 Da<br>[M+H]+: 443.097273 Da<br>[M-H]-: 441.08272 Da
Caffeine	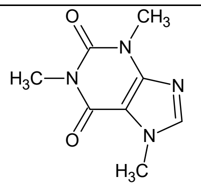	Molecular Formula: C8H10N4O2<br>Monoisotopic Mass: 194.080376 Da<br>[M+H]+ : 195.087652 Da<br>[M-H]- : 193.073099 Da

Sunphenon 90D is defined by the manufacturer as a decaffeinated green tea extract (see Resources - Sunphenon).

There are three main methods of decaffeination of coffee and tea:

1) Extraction of caffeine with organic solvents. Of these, the most effective are organochlorine solvents that are hazardous to health - chloroform, methylene chloride, tetrachloroethane and others. Less selective and effective are natural solvents such as ethyl acetate.

2) Extraction with water. The most complex method, but without harmful substances.

3) Extraction with supercritical fluids such as CO2. An effective, safe, but technically complex method. Its use allows you to achieve a high level of decoffeination.

The manufacturer claims that Sunphenon extracts are made from green tea leaves (Camellia sinensis), which are first extracted using a water infusion process. The extracts are then further decaffeinated using food grade ethyl acetate (it is unclear whether all or some of them).

Green tea is very rich in caffeine, richer than coffee, and extraction rarely allows for the complete removal of a component of a complex mixture, so a small content of caffeine is to be expected in the so-called decaffeinated extracts.

Note that the Sunphenon extract line contains more than 15 products, varying in technology and composition, which are disclosed by the manufacturer only in general terms. According to caffeine content, these products can be roughly divided into 3 groups: 1) with a very low caffeine content - less than 0.1%; 2) with a moderate caffeine content - less than 1%; 3) with a caffeine content of about 10% (obviously, these are not decaffeinated food-grade extracts). Examples of such extracts are given below:

Sunphenon EGCg®	EGCg > 94%, caffeine < 0.1%	High-purity decaffeinated EGCg for use in supplements.
Sunphenon 90D®	polyphenols > 90%, catechins > 80%, EGCg > 45%, caffeine < 1%	Decaffeinated catechins for use in supplements, foods and beverages with or without tea taste or color.
Sunphenon 90M-B®	polyphenols > 80%, catechins > 75%, EGCg > 40%, caffeine < 10%	High-purity catechins for use in supplements, foods and beverages with or without tea taste or color.

The purpose of the study was to identify and quantify the polyphenol and caffeine content of Sunphenon-90D green tea extract.

METHODS

HPLC-UV and HPLC-MS/UV methods were used to study the extracts.
- HPLC-MS was used to search and identify components of the extract.
- HPLC-UV was used to evaluate the content of individual detectable components in the substance under study using the method of normalization.

Search and identification of polyphenolic components by HPLC-MS.

Equipment.

For testing, a liquid chromatograph was used: Accela, Thermo scientific with a Thermo Scientific Accela PDA diode matrix detector and a Thermo Scientific TSQ Quantum Access MAX quadrupole detector, as well as a computer system for data collection and processing.

Preparation of solutions for chromatography.

Mobile phase component A: Place about 600 ml of deionized water in a 1000 ml volumetric flask and add 1 ml of formic acid. Bring the volume of solution to the mark with deionized water and mix.
Mobile phase component B: Place about 600 ml of acetonitrile in a 1000 ml volumetric flask and add 1 ml of formic acid. Bring the volume of solution to the mark with acetonitrile and mix.
Solvent: Deionized water.
Preparation of the test solution.
About 20.0 mg of the substance is placed in a 10 ml volumetric flask, dissolved in approximately 6 ml of solvent, then the volume of the solution is adjusted to the mark with the same solvent and mixed (the concentration of the substance in the solution is about 2.0 mg/ml).

Chromatographic conditions

Column	-	Agilent, Zorbax SB-C18 150x4.6 mm particle size 1.8 µm. An alternative column may be used that meets the suitability requirements of the chromatographic system.
Mode	-	Gradient
MP A	-	0.1% solution of formic acid in water
MP B	-	0.1% solution of formic acid in acetonitrile
Flow rate	-	1.0 ml/min
Column temperature	-	35 ºC
Sample volume	-	0.5 µl
Analysis time	-	25 minutes
Detector	-	mass spectrometer with quadrupole detector
Ionization method	-	electrostatic spray (HESI)
Atomizer voltage	-	3000 V
Ion source temperature	-	500 °C
Nebulizer gas pressure	-	60 psi
Dryer gas pressure	-	20 arbitrary units
Ion tube temperature	-	395 °C
Ion detection mode	-	TIC (50-1000 m/z)
Scan speed	-	0.800 scans per second

Gradient Mode

Time, min	Component A, %	Component B, %
0.0	99	1
1.0	99	1
15.0	75	25
18.0	5	95
20.0	5	95
20.1	99	1
25	99	1

MS chromatograms allow identification of the polyphenols by the mass of their molecular ion. UV chromatograms provide an idea of the retention time for subsequent assessment of the content of individual detectable components in the substance under study using the HPLC-UV method.

Equipment.

To carry out measurements, a liquid chromatograph with a diode matrix detector Nexera-i LC-2040C 3D Plus (Shimadzu) was used, as well as a computer system for collecting and processing data.

Preparation of solutions for chromatography.

Mobile phase component A: Place about 600 ml of deionized water in a 1000 ml volumetric flask and add 1 ml of trifluoroacetic acid. Bring the volume of solution to the mark with deionized water and mix.

Mobile phase component B: acetonitrile in required quantity.

Solvent: deionized water.

Preparation of the test solution.

About 60.0 mg of the substance is placed in a 10 ml volumetric flask, dissolved in approximately 6 ml of solvent, then the volume of the solution is adjusted to the mark with the same solvent and mixed (the concentration of the substance in the solution is about 6 mg/ml).

Chromatographic conditions

Column	-	Zorbax Eclipse Plus C18, 150 mm x 4.6 mm, 1.8 μm, Agilent. An alternative column may be used that meets the suitability requirements of the chromatographic system.
Mode	-	Gradient
MP A	-	0.1% solution of trifluoroacetic acid in water
MP B	-	Acetonitrile
Flow rate	-	1.0 ml/min
Column temperature	-	35 ºC
Sample volume	-	1-2 μl
Analysis time	-	35 minutes
Detector	-	diode matrix
Scan range	-	190-800 nm
Processing wavelength	-	275 nm

Gradient Mode:

Time, min	Component A, %	Component B, %
0.0	99	1
1.0	99	1
25.0	75	25
28.0	5	95
30.0	5	95
30.1	99	1
35.0	99	1

Calculation of component content.

The percentage content of any component (Xi) is calculated using the internal normalization method. The total content of components is calculated by summing the content of all individual impurities.

Not taken into account:
- peaks present in the solvent chromatogram (blank);
- system peaks.
The content of an individual component of the mixture is expressed as a percentage.

RESULTS

The search and identification of components of green tea extract was carried out using the HPLC-MS method, by searching mass chromatograms for the total ion current in the area of registration of positive ions and negative ions (Figure S1) for the masses of the corresponding molecular ions shown in Table S1.

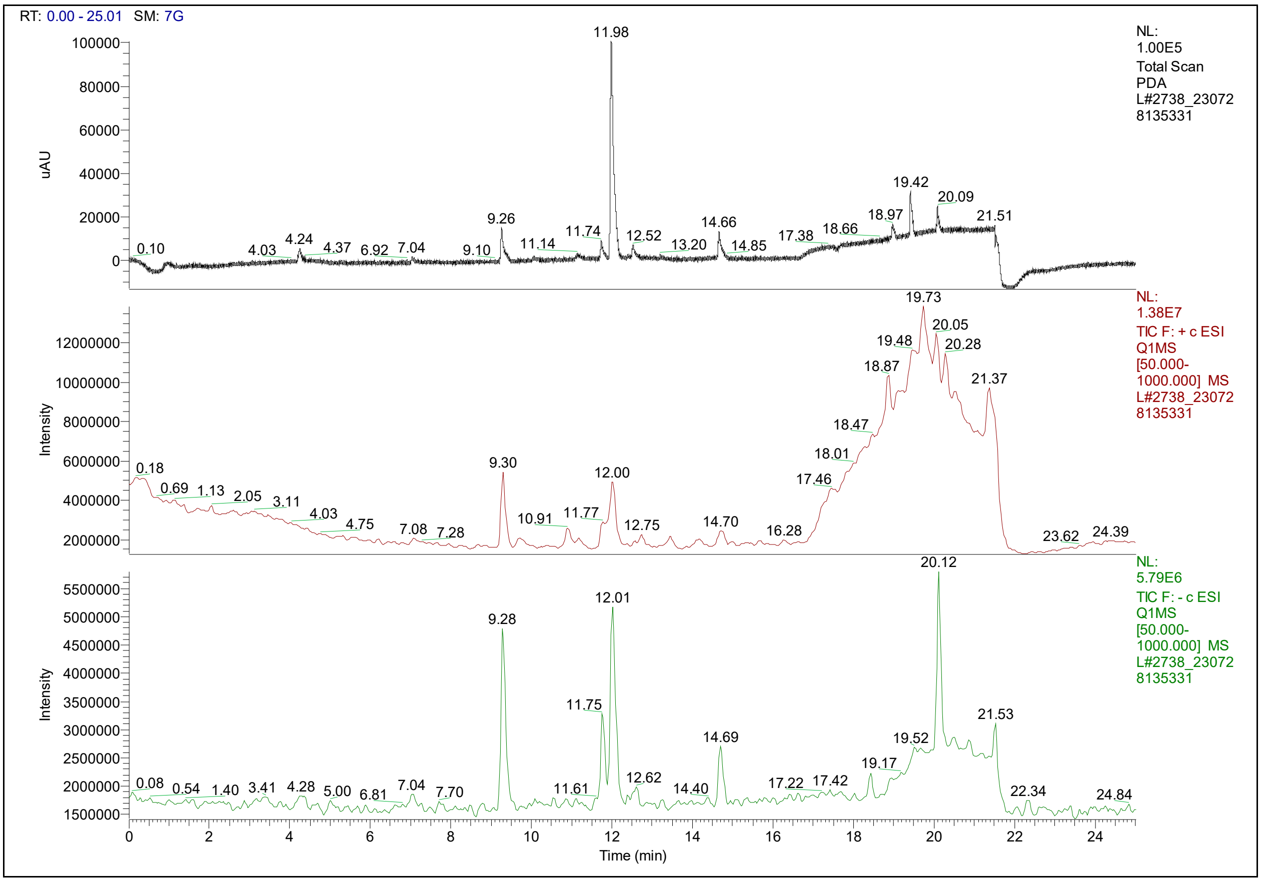

Figure S1. Extract chromatograms of Sunphenon 90D green tea extract by total ultraviolet absorption (in black), by total ion current in the region of registration of positive ions (in red), and by total ion current in the region of registration of negative ions (in green)

To estimate the content of individual components in the studied extracts based on HPLC-UV data, a normalization method was used, when the area of all peaks is taken as 100% and for related substances their content in the mixture is taken equal to the area of its chromatographic peak expressed as a percentage of the total area of all peaks. The results of assessing the content of each individual component of the mixture, expressed as a percentage, are given in Table S2.

The most pronounced picture of the separation of components was obtained on the mass chromatogram in the region of registration of negative ions (in green). Mass spectrometric characteristics allowing for the assignment of analytes are given in Table S3. The retention times on HPLC-UV are somewhat different from the retention times obtained by HPLC-MS, however, the data obtained allow us

to identify the main polyphenols without the use of standard samples by their single retention time to assess quantitative content.

Table S2 - Assessment of the content of extract components according to HPLC UV data

UV chromatogram of green tea extract Sunphenon-90D	Retention times and peak areas of individual components in the chromatogram

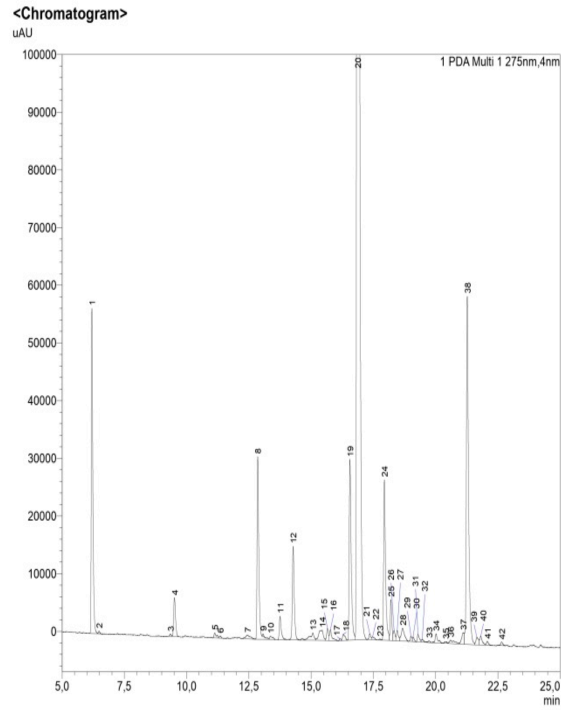

<Peak Table> L#2738_Sunphenon-90D_002.lcd

PDA Ch1 275nm

Peak#	Name	Ret. Time	Area	Area%
1	Галлат	6,196	238006	5,655
2		6,482	1824	0,043
3		9,348	1704	0,040
4		9,512	34951	0,830
5		11,128	5635	0,134
6		11,351	1923	0,046
7		12,443	8421	0,200
8	Эпигаллокатехин	12,857	154480	3,670
9		13,073	1414	0,034
10		13,366	4707	0,112
11		13,752	22037	0,524
12	Кофеин	14,279	85782	2,038
13		15,069	16406	0,390
14		15,442	22764	0,541
15		15,657	10422	0,248
16		15,775	12541	0,298
17		16,161	1487	0,035
18		16,295	7588	0,180
19	Эпикатехин	16,560	170176	4,043
20	Галлат эпигаллокатехина	16,868	2734369	64,965
21		17,357	4888	0,116
22		17,471	3303	0,078
23		17,761	833	0,020
24	Галлат эпикатехина	17,943	148448	3,527
25		18,204	30574	0,726
26		18,330	9016	0,214
27		18,459	10068	0,239
28		18,673	20509	0,487
29		19,010	4144	0,098
30		19,096	3702	0,088
31		19,294	7337	0,174
32		19,463	2774	0,066
33		19,734	1747	0,042
34		20,016	10983	0,261
35		20,405	2263	0,054
36		20,596	8528	0,203
37		21,107	12753	0,303
38		21,271	362749	8,618
39		21,658	9469	0,225
40		21,812	10902	0,259
41		22,076	4146	0,098
42		22,650	3195	0,076
Total			4208971	100,000

Table S3. Identified components of GTE and their contents

	Identifying Components from HPLC-MS Data		Component content according to UHPLC-UV data	
	RT	$[M+H]^+$ Ionization (positive)	RT	Contents in Sunphenon-90D, %
Epigallocatechin gallate	11.95 min	458.95 m/z;	16.85 min	64.96
Gallic acid (Gallate)	5.30 (4.24) min	н/a;	6.20 min	5.65
Epigallocatechin	9.30 min	306.96 m/z;	12.85 min	3.67
Epicatechin	11.70 min	290.90 m/z	16.56 min	4.04
Epicatechin gallate	14.65 min	442.98 m/z	17.94 min	3.53
Caffeine	10.91 min	195.01 m/z	14,28 min	2.04
Arachidic acid	30.12 min		21.20 min	8.62

In the extracts studied, all components contained in an amount of more than 1% were identified (except for a component with a retention time of 21.2 contained in the extracts in amounts of 8 and 25%, respectively, having a negatively charged molecular ion with a mass of 311.09). Molecular weight 312, taking into account the significant retention time, may correspond to a saturated carboxylic acid, for example, Arachidic acid (also known as Icosanoic acid), $C20H40O2$, found in coffee oils, cocoa and other products.

The amount of caffeine 2.04%, detected by the HPLC-UV screening method by the peak area relative to all components, generally corresponds to the order of its content declared by the manufacturer (caffeine < 1%). As well as the content of EGCg - declared > 45%, found 64.96%. The exact amount of caffeine can be determined using quantitative methods with calibration relative to standard samples (RM) of caffeine. Thus, the obtained data on the assessment of the caffeine content in decaffeinated green tea extract Sunphenon 90D do not contradict the data provided by the manufacturer.
